# Chassis selection and metabolic fine-tuning enable efficient in planta betalain production

**DOI:** 10.1093/plphys/kiag337

**Published:** 2026-06-08

**Authors:** Soyoung Jung, Marcos V V de Oliveira, Ray Collier, Abou Yobi, Ruthie Angelovici, Shawn M Kaeppler, Hiroshi A Maeda

**Affiliations:** Department of Botany, University of Wisconsin-Madison, Madison, WI, United States; Department of Botany, University of Wisconsin-Madison, Madison, WI, United States; Wisconsin Crop Innovation Center, University of Wisconsin-Madison, Middleton, WI, United States; Cristopher S. Bond Life Sciences Center, Division of Biological Sciences, Interdisciplinary Plant Group, University of Missouri, Columbia, MO, United States; Cristopher S. Bond Life Sciences Center, Division of Biological Sciences, Interdisciplinary Plant Group, University of Missouri, Columbia, MO, United States; Plant Biology and Department of Biochemistry and Molecular Biology, Michigan State University, East Lansing, MI, United States; Wisconsin Crop Innovation Center, University of Wisconsin-Madison, Middleton, WI, United States; Department of Botany, University of Wisconsin-Madison, Madison, WI, United States

## Abstract

Synthetic biology enables efficient production of valuable compounds in biological systems, including plants that capture atmospheric CO_2_ to synthesize and accumulate abundant and diverse specialized metabolites. Most plant synthetic biology studies to produce specialized metabolites have primarily used *Nicotiana benthamiana* as an underpinning metabolic chassis, due to its rapid agroinfiltration method, leaving much of the metabolic potential of other plant species underexplored. Here we engineered 3 distinct plant chassis—Arabidopsis, tobacco and soybean—by stably introducing an optimized betalain biosynthetic pathway and analyzed their metabolic impacts. Betalains are tyrosine-derived pigments, which are used as natural red and yellow food dyes with rapidly growing demand due to a recent regulatory shift. We fine-tuned metabolic balances by redesigning the *RUBY* betalain construct (*RUBY*v2), adding an extra DODA enzyme (“pull”) and modulating tyrosine precursor supply (“push”) using 2 different promoters. The “push-and-pull (push + pull)” lines produced higher betalain levels than the “pull” lines in all 3 species, even exceeding those of beet roots. While Arabidopsis and tobacco “push + pull” lines driven by a strong promoter showed dwarfism, corresponding soybean lines did not show severe growth defects, suggesting greater tolerance of soybean to the engineered pathway. This study demonstrates that careful plant chassis selection, coupled with precise control of pathway expression, is essential for maximizing the yield of target specialized metabolites, such as betalain pigments, without impairing overall plant growth.

## Introduction

Betalain pigments are tyrosine-derived specialized metabolites with yellow or red coloration and in plants are uniquely produced in members of the Caryophyllales order. In addition to their vivid hues, betalains exhibit potential health benefits including anticancer, anti-inflammatory, or antidiabetic activities (Gandía-Herrero et al. 2016; Khan 2016; Nirmal et al. 2024). Due to their high stability across a broad pH range, betalains are widely used as natural food colorants (Calva-Estrada et al. 2022; Carreón-Hidalgo et al. 2022; Martins et al. 2024). As of 2024, the global food dye market exceeds 4 billion USD (Towards FnB, 2025) and is largely dominated by petroleum-derived synthetic dyes. However, growing health and environmental concerns are accelerating the rapid shift toward safer, sustainable, natural alternatives. In January 2025, the US Food and Drug Administration (FDA) banned the use of synthetic dye Red No. 3 in food and ingested drugs (U.S. Food and Drug Administration 2025a), reflecting similar restrictions already in place across Europe and California. More recently, in April 2025, the FDA announced forthcoming regulations to phase out additional synthetic dyes, including Red No. 40, from the US food industry (US Food and Drug Administration 2025b). Despite increasing demand, natural betalains are much more expensive than synthetic counterparts, such as Red No. 40, largely due to the limited yield currently available from natural sources like beets (*Beta vulgaris*, Novais et al. 2022; Thomsen et al. 2023). Furthermore, cultivation of major sources of betalains, such as beets, is seasonal and cannot provide year-round supply, necessitating reliance on beet cultivation in other countries to meet continuous demands (Akan et al. 2021). These challenges underscore the urgent need for a scalable and sustainable production method for betalains.

Synthetic biology enables engineering of biological systems through iterative “design-build-test-learn (DBTL)” cycles (Nielsen and Keasling 2016; Pouvreau et al. 2018). This approach offers alternative bio-based production methods for a wide array of plant specialized metabolites by accelerating the metabolic engineering processes. Plants are emerging as promising and sustainable hosts for chemical production using synthetic biology. Although still in early stages, plant synthetic biology offers distinct advantages over microbial platforms (Barnum et al. 2021; Wang and Demirer 2023). Plants naturally synthesize and tolerate toxic compounds, possess complex pathways distributed across multiple cellular compartments, and have high storage capacities—especially in vacuoles that can occupy up to 90% of plant cells (Dünser et al. 2022; Xiao et al. 2025). In addition, plants grow by fixing atmospheric CO_2_ using sunlight energy and do not require costly fermentation infrastructure, reducing the need for external carbon inputs and operational costs. These advantages make plants an attractive platform for large-scale, sustainable production of valuable plant specialized metabolites.

Red beet roots, the primary commercial sources of betalains, typically accumulate 1.6 to 2.7 mg/g fresh weight (FW) or 10 to 17 mg/g dry weight (DW), of total betalains and 1.3 to 1.8 mg/g FW (8 to 14 mg/g DW) of betacyanins, the dominant red pigments (Sawicki et al. 2016). Betalain biosynthesis has been successfully reconstituted in multiple heterologous plant hosts. Polturak et al. (2017) generated stable pigment accumulation in Solanaceae crops—tomato, potato, and eggplant—with betacyanin levels of up to 0.12 mg/g FW in eggplant fruit and potato tuber and 0.25 mg/mL in tomato juice. The *RUBY* construct (He et al. 2020), which employs a polycistronic expression system, further simplified pathway engineering and enabled betalain pigmentation in multiple plant species, including carrot (0.94 mg/g DW, Deng et al. 2023) and *Nicotiana benthamiana* leaves (up to 1.03 mg/g FW, Pramanik et al. 2024).

To enhance the yield of betalains, subsequent strategies introduced the deregulated arogenate TyrA dehydrogenase from beets (BvTyrAα, Lopez-Nieves et al. 2018) to “push” tyrosine precursor supply together with expressing betalain genes. This approach enabled up to 15-fold increase in betalain accumulation, such as in tomato fruit (Grützner et al. 2021) and *N. benthamiana* leaves (Timoneda et al. 2018), compared with corresponding lines lacking BvTyrAα expression. More recently, the e*RUBY* construct was generated to polycistronically express 4 genes encoding BvTyrAα, cytochrome CYP76AD1 tyrosine hydroxylase, 3,4-dihydroxy-L-phenylalanine (L-DOPA) dioxygenase (DODA), and *cyclo*-DOPA 5-*O*-glucosyltransferase (*c*DOPA5GT). e*RUBY* expression resulted in betalain-producing rice endosperm up to 0.31 mg/g DW (Tan et al. 2025) and maize kernel up to 11.4 mg/g DW (Xue et al. 2026). Jung and Maeda (2024) further demonstrated that debottlenecking the DODA step, when combined with the enhanced tyrosine precursor supply, substantially increases betacyanin yields, reaching up to 2.9 mg/g FW in *N. benthamiana* leaves, surpassing levels typically found in beet roots.

One of the major challenges of plant synthetic biology stems from the high complexity of biosynthetic pathways (Sweetlove and Fernie 2013; Yang and Reyna-Llorens 2023). To reconstitute this complexity in heterologous plant hosts, fine-tuning the expression level of target genes is crucial to balance metabolic flux within the pathway and thus help increasing final yields (Rizzo et al. 2023; Wang and Demirer 2023). Yet, most plant synthetic biology studies, including the aforementioned betalain production, have relied on repetitive uses of a small set of “strong constitutive” promoters, rather than employing promoters with diverse strengths of expression as are observed in native plants. In addition, repeated use of identical promoters can increase the risk of recombination (Grützner et al. 2021; Golubova et al. 2024). Therefore, choosing right promoters is essential to accurately regulate target genes in heterologous plant hosts.

Selecting an appropriate plant chassis is also critical for optimizing heterologous compound production through synthetic biology, given the vast metabolic diversity that exists among plant species (Owen et al. 2017; Maeda 2019). Although primary metabolism is generally well conserved across plant lineages, certain variations exist and can affect precursor availability (Maeda 2019). For example, legumes possess an additional cytosolic tyrosine biosynthetic pathway beyond the canonical plastidial route, which may enhance the production of tyrosine-derived compounds including betalains (Schenck et al. 2015, 2017). However, most plant synthetic biology studies have relied on *N. benthamiana*, largely due to the ease of the Agrobacterium-mediated transient expression system, not necessarily because *N. benthamiana* has been validated as the most suitable production host. Chassis choice, however, must be treated as a core design parameter. Transformation ease alone does not ensure efficient metabolite production if the endogenous metabolism of a heterologous host cannot support engineered pathways. Therefore, evaluating species-specific precursor pools or metabolic tolerance can help identify hosts more capable of stable, high-level metabolite accumulation. Expanding plant synthetic biology beyond *N. benthamiana* to include diverse, stably transformable crops is essential for leveraging host-specific metabolic advantages and achieving scalable biosynthesis of valuable target compounds.

To address these limitations of plant synthetic biology, in terms of chassis selection and fine-tuning a synthetic pathway and its expression, this study engineered 3 distinct plant chassis—Arabidopsis, tobacco, and soybean—by stably transforming them with a series of DNA constructs. We used betalains as target compounds for developing and evaluating such engineering strategies given their relatively simple pathway, visual traceability, and commercial value with increasing demand due to both regulatory and consumer-driven shifts away from synthetic dyes (Polturak and Aharoni 2019; Khan and Polturak 2025). Here we aimed to enhance betalain production by combining increased supply of tyrosine precursor (“push”) with a debottlenecking DODA step (“pull”). Data obtained from all of the generated transgenic lines, including ones with negative results, are shown in Data S1. Relative to the “pull” lines across all 3 species, the “push + pull” lines exhibited substantially higher betacyanins levels, which surpassed the concentration of betacyanins typically accumulated in red beet roots. While these results were compelling, the Arabidopsis and tobacco “push + pull” lines showed severe growth defects, likely due to elevated accumulation of tyrosine and the intermediate L-DOPA. Remarkably, however, soybean “push + pull” lines still maintained robust growth despite similarly high metabolite levels. These findings suggest that soybean, relative to Arabidopsis and tobacco, is more tolerant to integration and engineering of a tyrosine-derived specialized metabolite synthesis pathway, highlighting its potential as a robust and scalable chassis for betalain production. Furthermore, we demonstrated the importance of promoter choice for fine-tuning “push” expression for maintaining high pigment production while concurrently mitigating growth defects. Our results underscore both the importance of plant chassis selection as well as the need for precise regulation of transgene expression in synthetic biology efforts aimed at optimizing heterologous production of valuable plant specialized metabolites.

## Results

### The initial “push/pull” construct failed to generate stable tobacco and soybean transgenic lines

**Figure 1:**
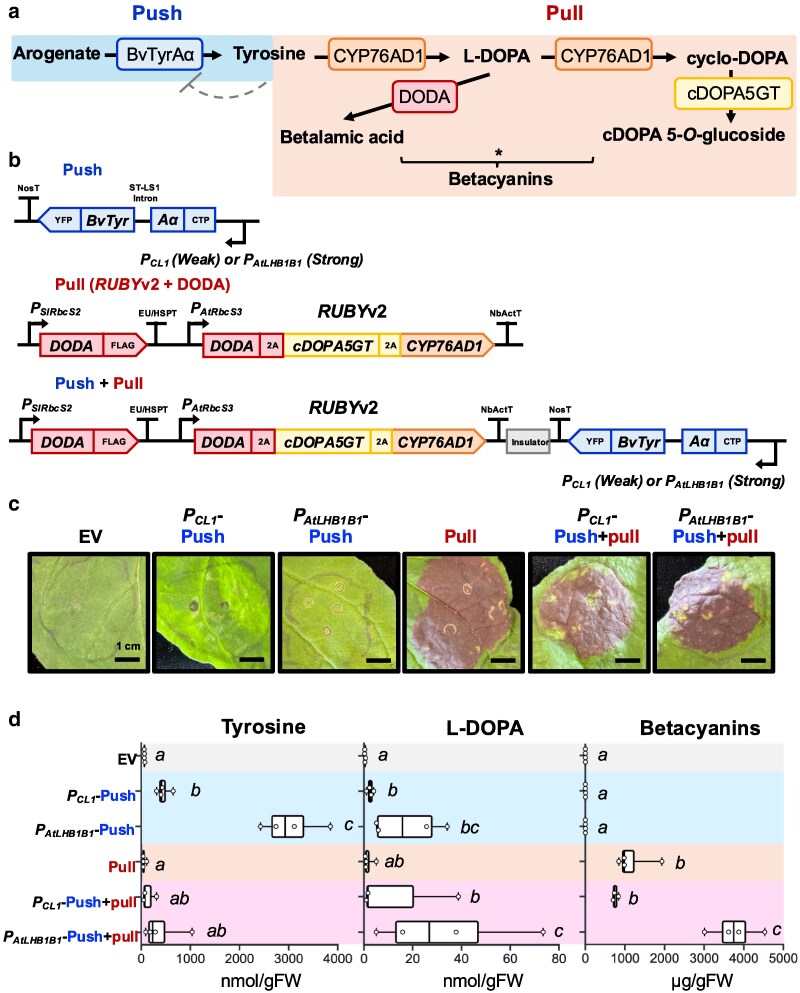
Optimized betalain construct with additional tyrosine supply boosts betalain production in *N. benthamiana* leaves. a) A simplified biosynthetic pathway of betalain pigments. Arogenate dehydrogenase from beet (BvTyrAα) shows relaxed feedback inhibition by tyrosine and was used to “push” the supply of tyrosine in this study. The betalain biosynthetic pathway was used to “pull” the enhanced tyrosine supply for producing betalains. Cytochrome P450 CYP76AD1 catalyzes the first step to convert tyrosine to L-DODA, which is then used to form betalamic acid catalyzed by L-DOPA 4,5-dioxygenase (DODA) or *cyclo*-DOPA (cDOPA) by CYP76AD1. cDOPA 5-*O*-glucosyltransferase (*c*DOPA5GT) glucosylates cDOPA to generate cDOPA 5-*O*-glucoside. Asterisk denotes a spontaneous reaction to form betacyanins. b) Schematic diagrams of “push,” “pull,” and “push + pull” constructs. Either Arabidopsis light-harvesting chlorophyll–protein complex II subunit B1 (*P_AtLHB1B1_*) or Arabidopsis NADH dehydrogenase ubiquinone 1 beta subcomplex subunit promoter (*P_CL1_*) and nopaline synthase terminator (NosT) were used for expressing BvTyrAα coding sequence flanked with N-terminal petunia chloroplast transit peptide (CTP) and C-terminal YFP tag. The second intron sequence of potato *ST-LS1* gene was inserted in the coding sequence of BvTyrAα to prevent marginal expression of “push” construct in prokaryotes; tomato rubisco promoter (*P_SlRbcS2_*) and *N. benthamiana* Actin3 terminator (NbActT) were used to express *RUBY*v2. Arabidopsis rubisco promoter (*P_AtRbcS3_*) and intronless Tobacco Extensin terminator (EU) in combination with a Heat Shock Protein terminator (EU/HSPT) were used for expressing BvDODA coding sequence flanked with C-terminal FLAG tag. c) Agrobacterium-mediated transient expression of constructs in *N. benthamiana* leaves. Photographs taken at 3 d postinfiltration (dpi). d) Quantification of tyrosine, L-DOPA, and betacyanin levels in infiltrated leaves. Tyrosine and L-DOPA levels were analyzed by LC-MS. Total betacyanin content was measured by absorbance at 538 nm using a spectrophotometer. The experiment was conducted using 4 biological replicates. Letters denote significant differences based on 1-way ANOVA of log_10_-transformed values followed by Tukey's HSD test (*P* < 0.05). EV, empty vector. Box plots were generated in BioRender (https://www.biorender.com).

To test whether the “push-and-pull” strategy enhances betalain production in heterologous plant hosts (Fig. 1a), 4 initial constructs were generated to express either (i) an empty vector (EV), (ii) enhanced tyrosine production through overexpression of *BvTyrAα* (“push”), (iii) 3 betalain genes (“pull”), or (iv) *BvTyrAα* and 3 betalain genes (“push/pull”; Figure S1a, Jung and Maeda 2024). Because the metabolic bottleneck in the betalain pathway had not yet been identified, our initial design used the constitutive promoters, which enable continuous expression without tailoring expression levels of individual genes. Tobacco and soybean, among the 3 species, were initially used for stable transformation, as betalain pigmentation could be visually detected during the early stages of tissue culture (eg, callus and shoot regeneration, or emerging meristematic tissues and early shoots). Transgenic *T*_0_ tobacco lines were successfully generated from the EV, push, and pull constructs; however, no stable transgenic lines were obtained with the push/pull construct (Figure S1b). Similarly, in soybean, while multiple stable *T*_0_ lines were generated for EV, push, and pull constructs, no push/pull lines were obtained (Data S1). Sequencing confirmed that there were no recombinations or errors within the binary vector constructs. Given the consistent failure in both tobacco and soybean, these constructs were not tested in Arabidopsis.

To investigate the underlying cause of the unsuccessful outcome, these constructs were transiently expressed in *N. benthamiana* and a series of experiments were carried out, as we reported earlier (Jung and Maeda 2024). The “push/pull” constructs showed limited conversion from L-DOPA to betalains, resulting in hyperaccumulation of L-DOPA. The highest betalain production was achieved when the *RUBY* “pull” construct (He et al. 2020) was co-expressed with the “push” and an additional copy of the *DODA* gene (Jung and Maeda 2024). These results suggest that an imbalance between “push” and “pull” engineering leads to excess L-DOPA and/or its derivatives, which likely interfered with successful generation of stable transgenic lines.

### New betalain constructs to balance push and pull for optimized betalain production

To further alleviate the bottleneck step catalyzed by the DODA enzyme, a new *RUBY*v2 construct was designed by rearranging the betalain biosynthetic genes to place *DODA* upstream of the other 2 betalain genes (Fig. 1b). This design leverages the assumed higher translational efficiency and expression level of the first gene in polycistronic expression systems (de Felipe et al. 2006; Jiao et al. 2018). Additionally, an extra *DODA* transcriptional unit was included in the new “pull” construct (Fig. 1b). To prevent possible recombination between 2 identical sequences, we used a *B. vulgaris BvDODA* transcriptional unit with a distinct DNA sequence from the codon-optimized *BvDODA* in the *RUBY* construct, encoding the same BvDODA protein.

We also inserted the potato ST-LS1 intron sequence containing an in-frame premature stop codon (Eckes et al. 1986; Vancanneyt et al. 1990; Brophy et al. 2022) in the *BvTyrAα* coding sequence of the new “push” and “push + pull” constructs (Fig. 1b). This is to allow expression of functional *BvTyrAα* only *in planta*, as our prior work suggested that leaky *BvTyrAα* transgene expression in Agrobacterium might compromise the bacterial viability and transformation efficiency (Jung and Maeda 2024). Additionally, *BvTyrAα* was expressed under 2 different promoters to find optimal levels of the “push” effect. The *P_AtLHB1B1_* promoter from Arabidopsis light-harvesting chlorophyll–protein complex II subunit B1 (AT2G34430, Engler et al. 2014) was used to express *BvTyrAα* (“*P_AtLHB1B1_*-push”) at high levels and in same photosynthetic tissues with the new “pull” construct where 2 rubisco promoters (ie, *P_AtRbcS3_* and *P_SlRbcS2_*) were used to express *RUBY*v2 and *BvDODA*. We also used a weak constitutive *P_CL1_* promoter from Arabidopsis NADH dehydrogenase ubiquinone 1 beta subcomplex subunit (AT1G76200) for expressing *BvTyrAα* (“*P_CL1_*-push”, Zhou et al. 2023, Fig. 1b). Accordingly, 2 new “push + pull” constructs were generated (ie, “*P_AtLHB1B1_*-push + pull” and “*P_CL1_*-push + pull”), each expressing *BvTyrAα*, *RUBY*v2, and an additional *BvDODA* (Fig. 1b).

The functionality of the newly generated constructs was first tested by transiently expressing them in *N. benthamiana* leaves. No visible pigmentation was observed in leaves expressing EV, *P_AtLHB1B1_*-push, or *P_CL1_*-push constructs as expected. In contrast, strong pigmentation was observed in pull-, *P_AtLHB1B1_*-push + pull-, or *P_CL1_*-push + pull-expressing leaves (Fig. 1c). The infiltration spots were harvested at 3 days post-infiltration (dpi) and subjected to metabolite profiling using liquid chromatography-mass spectrometry (LC–MS). The levels of tyrosine were the highest in *P_AtLHB1B1_*-push expressing leaves, followed by *P_CL1_*-push, reflecting the different expression levels between these 2 promoters (Fig. 1d). The push + pull-expressing leaves had tyrosine levels slightly or significantly lower than their corresponding push-only lines, suggesting that tyrosine was utilized for the downstream betalain production (Fig. 1d). L-DOPA levels in these push + pull-expressing leaves were detected at much lower levels than in those expressing the previous push/pull construct (Figure S1a, Jung and Maeda 2024), indicating enhanced efficiency in converting L-DOPA to betalains in the new pull construct that rearranged the order in the *RUBY*v2 and added an additional DODA transcriptional unit (Fig. 1d). When the betacyanins content was quantified using a spectrophotometer, betacyanin levels in *P_CL1_*-push + pull-expressing leaves were slightly lower than in pull-expressing leaves. The highest betacyanin levels were achieved in the *P_AtLHB1B1_*-push + pull-expressing leaves, up to 4.54 mg/g FW (8.25 *μ*mol/g FW) with the average of 3.77 mg/g FW, even exceeding betacyanin production in beet roots/hypocotyls (1.29 to 1.84 mg/g FW, Sawicki et al. 2016, Fig. 1d). These results validated that these new “push + pull” constructs produced high levels of betacyanins, without substantial accumulation of L-DOPA in *N. benthamiana* leaves.

### Tobacco *T*_0_ lines with the *P_CL1_*-push + pull construct showed high betacyanin levels without a plant growth penalty

**Figure 2:**
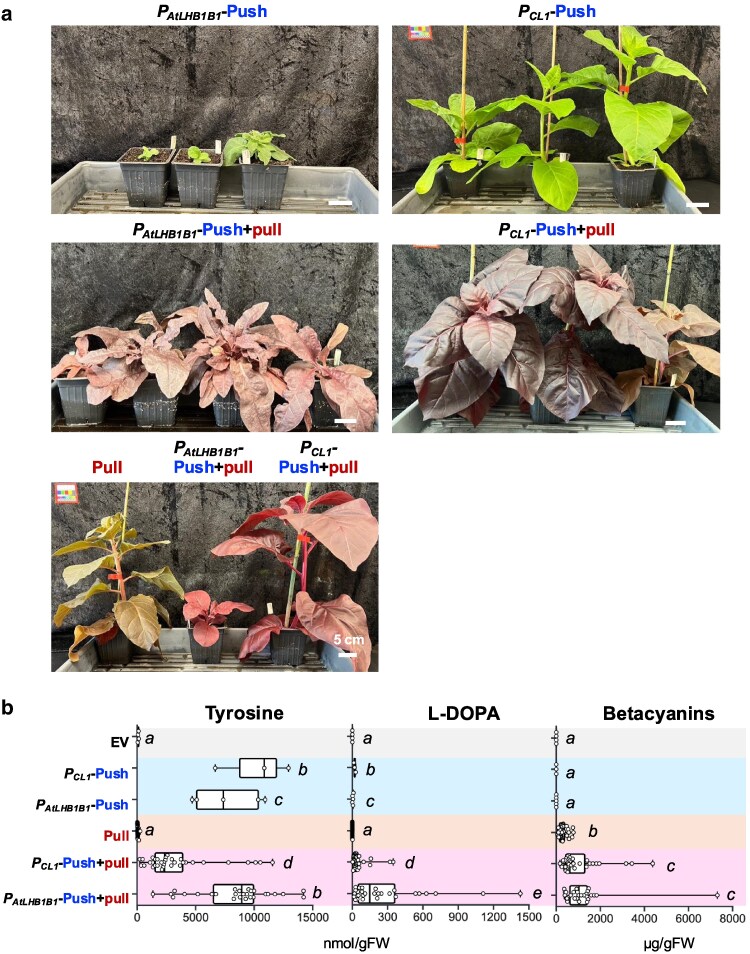
Push + pull construct partially alleviates growth penalty seen in *T*_0_ tobacco push lines. a) Phenotypes of stable *T*_0_ tobacco plants expressing push, pull, or push + pull constructs at 6 wk after transplanting from rooting media to soil. Push constructs used either *P_AtLHB1B1_* (strong constitutive) or *P_CL1_* (weak constitutive) promoter. b) Quantification of tyrosine, L-DOPA, and betacyanin levels in mature leaves of stable tobacco transgenic *T*_0_ lines. Leaf discs were collected at 6 wk after transplanting from rooting media to soil for metabolite analysis. Tyrosine and L-DOPA levels were analyzed by LC–MS. Total betacyanin content was measured by absorbance at 538 nm using a spectrophotometer. The experiment was conducted using biological replicates, with sample sizes as follows: EV (*n* = 4), *P_CL1_*-push (*n* = 3), *P_AtLHB1B1_*-push (*n* = 5), pull (*n* = 32), *P_CL1_*-push + pull (*n* = 34), and *P_AtLHB1B1_*-push + pull (*n* = 28). Letters denote significant differences based on 1-way ANOVA of log_10_-transformed values followed by Tukey's HSD test (*P* < 0.05). EV, empty vector. Box plots were generated in BioRender (https://www.biorender.com).

To evaluate whether the modified betalain constructs could be used to generate stable transgenic plants, Agrobacterium-mediated stable transformation was performed in tobacco (*Nicotiana tabacum* L. cv. “Petit Havana” SR1). In contrast to earlier results (Figure S1b), stable *T*_0_ lines were successfully obtained for all constructs (ie, EV, *P_AtLHB1B1_*-push, *P_CL1_*-push, pull, *P_AtLHB1B1_*-push + pull, and *P_CL1_*-push + pull, Fig. 2a). Betalain pigmentation was visible in stems, leaves, roots, and flowers of the tobacco lines expressing pull, *P_AtLHB1B1_*-push + pull, and *P_CL1_*-push + pull constructs, with pigments primarily localized in the central vacuoles of mesophyll cells (Figure S2). Both push + pull constructs enhanced pigmentation intensity relative to the pull construct (Fig. 2a), indicating that increased precursor supply boosts betalain production with the new betalain construct in tobacco. Transgenic lines expressing the *P_AtLHB1B1_*-push construct exhibited severely stunted growth and curled leaves; however, these growth defects were partially alleviated in *P_AtLHB1B1_*-push + pull lines, showing improved growth, larger leaves, and strong betalain pigmentation despite residual dwarfism (Fig. 2a, Data S1). Lines expressing *P_CL1_*-push construct showed a milder growth penalty than the *P_AtLHB1B1_*-push construct, including slightly reduced height and a reticulate leaf pattern. The *P_CL1_*-push + pull lines exhibited no observable growth defects despite their strong pigmentations (Fig. 2a, Data S1). These results revealed that modulating *BvTyrAα* “push” expression through promoter selection can influence the severity of growth penalties, which can be further mitigated by expressing the pull construct that redirects the accumulated tyrosine toward betalain biosynthesis.

To assess metabolic changes in these transgenic tobacco lines, leaf tissues were harvested 6 week after transplanting from rooting media to soil and metabolites were extracted. Both push + pull lines showed elevated betacyanin accumulation, reaching up to 7.31 mg/g FW (13.27 *μ*mol/g FW) with the average of 1.23 mg/g FW in the *P_AtLHB1B1_*-push + pull line and 4.38 mg/g FW (7.95 *μ*mol/g FW) with the average of 1.01 mg/g FW in the *P_CL1_*-push + pull line, exceeding betacyanin levels typically found in beet roots/hypocotyls (Fig. 2b). Tyrosine levels were significantly higher in both push and push + pull constructs compared with the EV control (Fig. 2b), consistent with the results from transient expression assays (Fig. 1d). Between the 2 push + pull lines, *P_CL1_*-push + pull exhibited significantly lower tyrosine levels than the *P_AtLHB1B1_*-push + pull. Likewise, L-DOPA levels were significantly lower in the *P_CL1_*-push + pull lines compared with the *P_AtLHB1B1_*-push + pull lines (Fig. 2b). These differences in tyrosine and/or L-DOPA may explain the growth defect observed only in the *P_AtLHB1B1_*-push + pull lines, not in *P_CL1_*-push + pull lines, as their high accumulation may be detrimental to the plant (Soares et al. 2014; Schenck and Maeda 2018; de Oliveira et al. 2019).

Tyramine is a decarboxylated derivative of tyrosine. Tyramine levels were significantly increased in both push and push + pull lines compared with the EV control and were significantly reduced in push + pull lines relative to push-only lines (Figure S3). This result suggests that excess tyrosine is successfully directed toward betalain production rather than toward tyramine biosynthesis when the betalain gene expression is carefully controlled. The levels of dopamine, a decarboxylated L-DOPA, were the highest in *P_AtLHB1B1_*-push + pull followed by *P_CL1_*-push + pull but overall remained much lower than tyramine (Figure S3). Further untargeted metabolomics analysis predicted that tyrosine derivatives such as homogentisate (HGA), an intermediate of the tyrosine-degradation pathway, as well as some phenylpropanoids (eg, *p*-coumarate) were decreased, while L-DOPA-derived compounds (eg, norepinephrine) were likely elevated in push + pull lines compared with push lines (Figure S4, Data S2).

RT-qPCR analysis of the same leaf samples showed that transcript levels of the betalain pathway genes were similar or even higher in the pull lines compared with the push + pull lines, yet the push + pull lines accumulated substantially higher levels of betalains (Figures S5 and S6). This indicates that increased precursor availability, rather than differences in betalain gene expression, drives the elevated pigment production in the push + pull lines. Furthermore, *BvTyrAα* transcripts were detected only in lines expressing push or push + pull constructs as expected, with *P_AtLHB1B1_*-driven lines exhibiting higher transcript abundance than their *P_CL1_*-driven counterparts (Figures S5 and S6), consistent with the expected difference in promoter strength. Taken together, these results demonstrate that enhanced precursor availability contributes to the efficient production of betalains in the push + pull lines.

### Transgenic *T*_0_ soybean lines showed higher tolerance than tobacco against elevated tyrosine and L-DOPA levels

**Figure 3:**
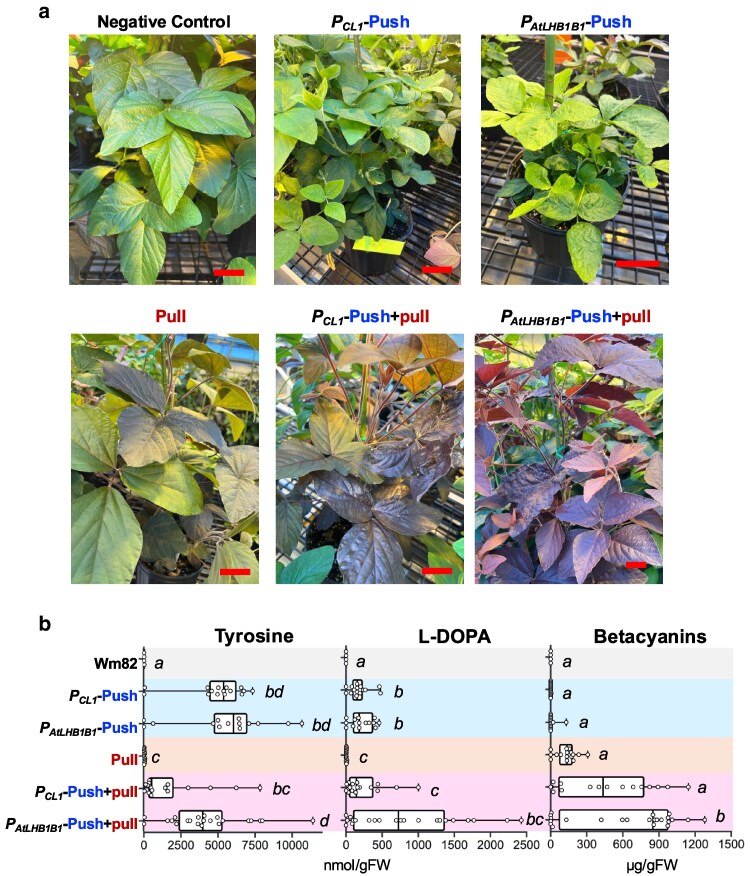
Soybean *T*_0_ transgenic lines show higher tolerance to tyrosine and L-DOPA than transgenic tobacco lines. a) Phenotypes of stable *T*_0_ soybean lines expressing push, pull, or push + pull constructs; Negative control, nontransformant line from tissue culture. b) Quantification of tyrosine, L-DOPA, and betacyanin in mature leaves of soybean *T*_0_ stable transgenic lines. Tyrosine and L-DOPA levels were analyzed by LC–MS. Total betacyanin content was measured by absorbance at 538 nm using a spectrophotometer. The experiment was conducted using biological replicates, with sample sizes as follows: Wm82 (*n* = 4), *P_CL1_*-push (*n* = 15), *P_AtLHB1B1_*-push (*n* = 14), pull (*n* = 15), *P_CL1_*-push + pull (*n* = 16), and *P_AtLHB1B1_*-push + pull (*n* = 22). Letters denote significant differences based on 1-way ANOVA of log_10_-transformed values followed by Tukey's HSD test (*P* < 0.05). Wm82, Williams 82. Box plots were generated in BioRender (https://www.biorender.com).

To evaluate metabolic impacts of expressing the modified constructs across different plant species, the same set of constructs was also introduced into soybean (*Glycine max*), which has an additional cytosolic tyrosine biosynthetic pathway (Schenck et al. 2015, 2017). As in tobacco, stronger pigmentation was observed in both soybean *P_AtLHB1B1_*- and *P_CL1_*-push + pull lines compared with the pull lines (Fig. 3a). Both push lines exhibited slightly stunted growth, which became more pronounced at the later developmental stages. Notably, unlike tobacco, none of the push + pull lines showed severe growth penalties, even in the *P_AtLHB1B1_*-push + pull soybean *T*_0_ lines (Fig. 3a). Both push + pull soybean *T*_0_ lines were able to finish their life cycle, although they showed reduced seed yields (Figure S7).

Betacyanin accumulation was slightly or significantly higher in the leaves of both “push + pull” lines relative to the “pull” lines, consistent with the visual phenotype. The highest betacyanin level was 1.28 mg/g FW (2.32 *μ*mol/g FW) with the average of 0.62 mg/gFW in a *P_AtLHB1B1_*-push + pull line, comparable to levels in beet roots/hypocotyls (Fig. 3b). The leaves of both push and push + pull lines accumulated significantly increased tyrosine levels compared with its wild-type Williams 82 (Wm82). The highest L-DOPA levels were observed in *P_AtLHB1B1_*-push + pull, followed by *P_CL1_*-push + pull lines, with levels reaching up to 2.23 nmol/g FW (440 *μ*g/g FW) in a *P_AtLHB1B1_*-push + pull, substantially exceeding those observed in transgenic tobacco (Fig. 3b). Similar to the results shown in tobacco *T*_0_ lines, untargeted metabolite analysis showed that tyrosine, tyrosine-derived compounds, and phenylpropanoids were decreased, whereas L-DOPA and its derivatives were elevated in push + pull lines compared with push-only lines (Figure S4, Data S3). Given that these soybean push + pull lines exhibited high L-DOPA and betacyanin levels without a severe growth penalty, soybean may possess greater tolerance to L-DOPA and its derivatives and thus could serve as a platform with greater flexibility with a wider dynamic range for betalain production than might be possible in tobacco.

RT-qPCR revealed higher *BvTyrAα* expression in the *P_AtLHB1B1_*-push or *P_AtLHB1B1_*-push + pull lines than in their *P_CL1_*-driven counterparts as expected. Betalain biosynthetic gene expression did not significantly differ between push + pull or pull constructs in soybean (Figures S8 and S9), consistent with the results observed in tobacco. We then selected *T*_0_ lines with a single-copy insertion, analyzed by digital PCR (Collier et al. 2017, Data S1), and harvested their seeds for further propagation. While both push-only lines produced almost no seeds (Figure S7), the push + pull lines produced substantial numbers of seeds (average of 123 and 249 for *P_CL1_*- and *P_AtLHB1B1_*-push + pull lines, respectively), though they remained lower than those of EV or pull lines (average of 290 or 337 seeds per plant respectively, Figure S7). These results suggest that redirecting the accumulated tyrosine toward betalain production by expressing the pull construct can mitigate compromised seed yields in soybean push lines.

### Betalain-producing Arabidopsis lines exhibited severe growth defects, did not produce seeds, or failed to germinate

The new constructs (Fig. 1b) were also introduced to Arabidopsis to further evaluate cross-species transformability of the betalain pathway engineering. *T*_1_ seeds were collected following floral dipping and screened for a seed-specific RFP marker expression. Only RFP-positive *T*_1_ seeds were selected for germination. Both push lines showed typical tyrosine-hyperaccumulating phenotypes (de Oliveira et al. 2019), including reticulate leaves and stunted growth (Figure S10a). *T*_1_ pull lines exhibited betalain pigmentation without any noticeable growth defects. Notably, floral dipping with push + pull constructs yielded only 2 RFP-positive seeds per construct, significantly fewer than with other constructs. However, these RFP-positive *P_AtLHB1B1_*-push + pull *T*_1_ lines showed severe growth defects and failed to produce seeds (Figure S10a). One of the RFP-positive *P_CL1_*-push + pull *T*_1_ lines showed a similarly severe growth phenotype and sterility, while the other grew normally with strong pigmentation. However, pigmentation intensity could not be clearly distinguished from that of the pull lines, unlike observations in tobacco or soybean (Figure S10a).

In the *T*_2_ generation, the *P_AtLHB1B1_*-push lines continued to exhibit pronounced growth defects and reticulate leaf phenotype, whereas no *T*_2_ seeds from *P_CL1_*-push lines germinated (Figure S10b). Similarly, *P_CL1_*-push + pull *T*_2_ seeds failed to germinate. Unexpectedly, some pull *T*_2_ lines showed severe growth defects (Figure S10b), despite the lack of noticeable phenotype in their *T*_1_ lines. This may be due to the homozygosity of the transgenes in their *T*_2_ generation. Therefore, Arabidopsis lines producing high levels of betalains often exhibit severe growth defects, sterility, or compromised seed viability, suggesting that Arabidopsis may not be a suitable plant host for betalain production.

### Strong pigmentation in push + pull lines is heritable in tobacco *T*_1_ generation

**Figure 4:**
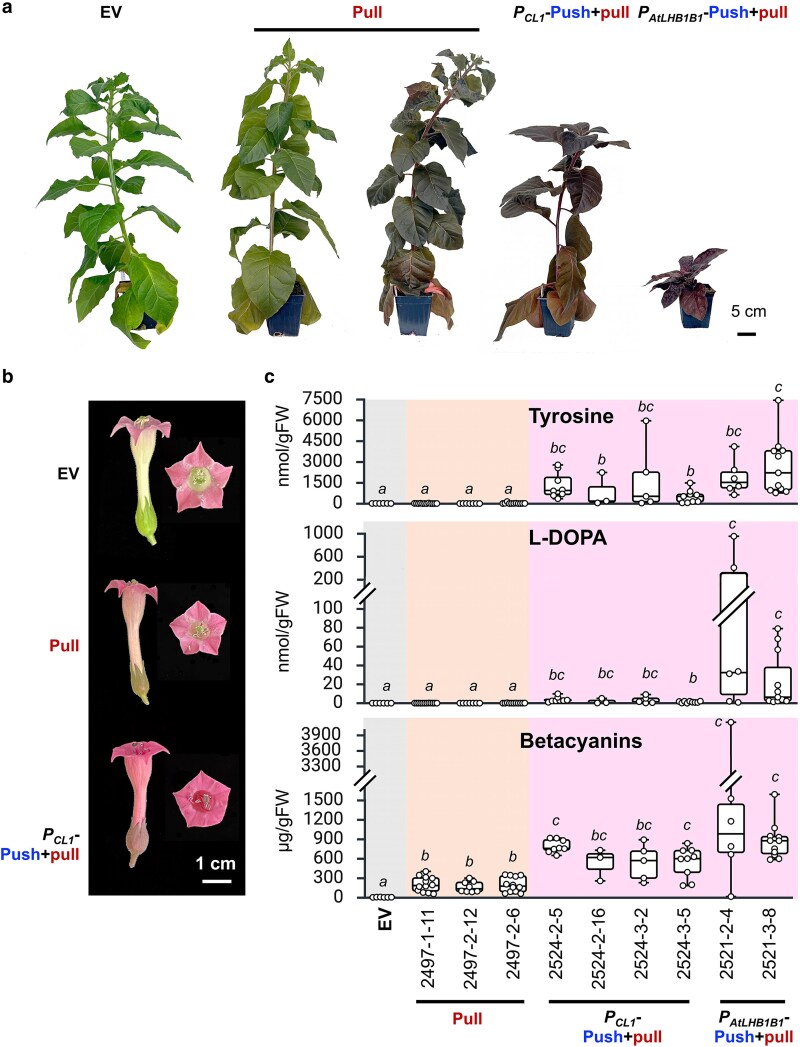
Betalain pigments are heritable in tobacco *T*_1_ lines. a) Phenotypes of stable *T*_1_ tobacco lines expressing EV, pull, or push + pull constructs at 3 mo after germination. EV, empty vector. b) Phenotypes of flowers from stable *T*_1_ tobacco lines of EV, pull, or *P_CL1_*-push + pull constructs. c) Quantification of tyrosine, L-DOPA, and betacyanin levels in mature leaves of tobacco stable transgenic *T*_1_ lines. Tyrosine and L-DOPA levels were analyzed by LC–MS. Total betacyanin content was measured by absorbance at 538 nm using a spectrophotometer. The experiment was conducted using biological replicates, with sample sizes as follows: EV (*n* = 6), 2497-1-11 (*n* = 12), 2497-2-12 (*n* = 8), 2497-2-6 (*n* = 12), 2524-2-5 (*n* = 8), 2524-2-16 (*n* = 3), 2524-3-2 (*n* = 5), 2524-3-5 (*n* = 9), 2521-2-4 (*n* = 6), and 2521-3-8 (*n* = 11). Letters denote significant differences based on 1-way ANOVA of log_10_-transformed values followed by Tukey's HSD test (*P* < 0.05). Box plots were generated in BioRender (https://www.biorender.com).

To determine whether betalain production is heritable in tobacco, *T*_1_ seeds harvested from *T*_0_ plants were assessed for segregation using seed-specific RFP signals. Only *T*_1_ lines showing a 3:1 ratio of RFP positive:negative were classified as single-copy insertions and selected for germination (Data S1). Because *P_AtLHB1B1_*-push + pull *T*_0_ lines exhibited growth defects, *T*_1_ seeds were available from only 2 *P_AtLHB1B1_*-push + pull lines and were germinated without copy number selection. Consistent with the previous generation, both push + pull lines exhibited stronger pigmentation than pull lines in the *T*_1_ generation, and *P_AtLHB1B1_*-push + pull *T*_1_ plants continued to display delayed development and smaller size compared with EV, with severity increased as the plants matured (Fig. 4a). In contrast, *P_CL1_*-push + pull lines of tobacco successfully germinated, unlike in the corresponding Arabidopsis lines, and exhibited only slightly delayed development relative to EV while overall maintaining a normal growth phenotype with high betalain pigmentation in all tissues (Fig. 4a, b). Total betacyanin levels were higher in leaves of both push + pull lines compared with pull-only lines (Fig. 4c). Targeted leaf metabolite analysis further revealed elevated levels of tyrosine and L-DOPA in *P_AtLHB1B1_*-push + pull lines compared with *P_CL1_*-push + pull lines (Fig. 4c). These results suggest that promoter choice can impact the levels of precursor, intermediates, and their derivatives as well as host growth outcome.

### Soybean *T*_1_ lines maintain high betalain levels by redirecting metabolic flux toward the tyrosine-derived pathway with minimal growth penalty

To evaluate the heritability of soybean transgenics, *T*_1_ seeds derived from single-copy *T*_0_ lines were germinated, and digital PCR was conducted to determine their zygosity (Data S1). In the *T*_1_ pull lines, homozygous individuals exhibited stronger pigmentation in the hypocotyls at early germination compared with hemizygous lines (Figure S11a). However, as the plants developed, pigmentation in homozygous pull lines gradually faded, resulting in a patchy pigmentation phenotype in the leaves, which was not observed in hemizygous lines (Figure S11b). This contrast became more pronounced at maturity, with homozygous pull lines displaying a near wild-type phenotype with green leaves and pods, whereas hemizygous lines retained betalain pigmentation throughout vegetative and reproductive tissues (Figure S11c). This may be attributed to ribosome stalling and No-Go RNA Decay due to the 3-consecutive-histidine motif within the *c*DOPA5GT coding sequence (Kramer et al. 2025).

**Figure 5:**
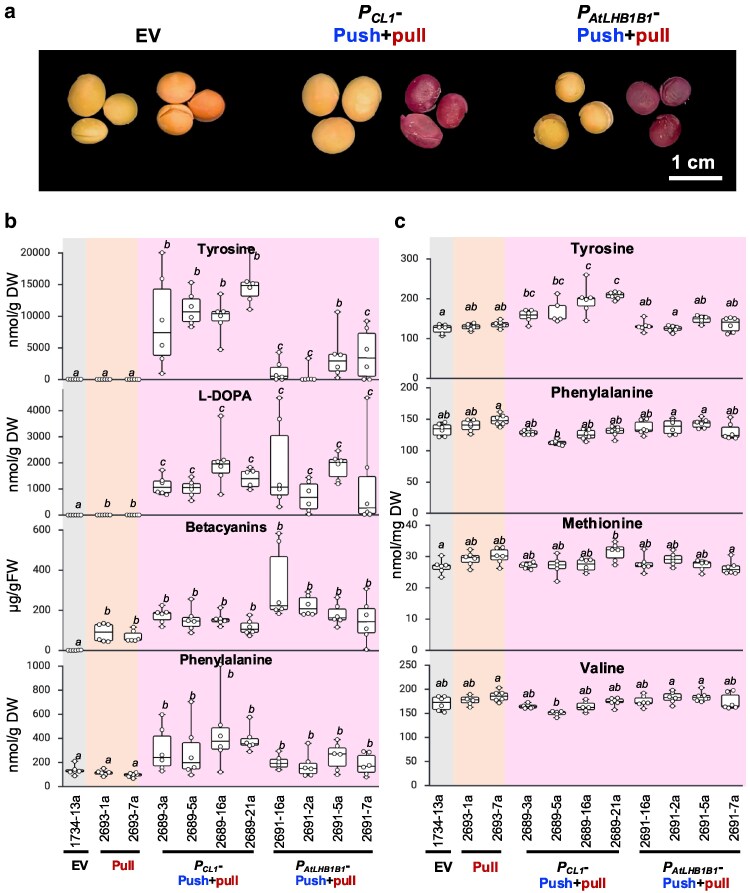
*P_CL1_*-push + pull *T*_1_ soybean seeds show elevated free and protein-bound tyrosine levels. a) Phenotypes of *T*_1_ soybean seeds from EV, *P_CL1_*-push + pull and *P_AtLHB1B1_*-push + pull lines, shown with and without seed coats. b) Quantification of free tyrosine, L-DOPA, betacyanin, and phenylalanine levels in transgenic soybean *T*_1_ seeds. Tyrosine and L-DOPA levels were analyzed by LC–MS. Total betacyanin content was measured by absorbance at 538 nm using a spectrophotometer. The experiment was conducted using 6 biological replicates. Letters indicate significant differences based on 1-way ANOVA of log_10_-transformed values followed by Tukey's HSD test (*P* < 0.05). c) Quantification of protein-bound amino acid levels in soybean transgenic *T*_1_ seeds. The experiment was conducted using 6 biological replicates. Letters indicate significant differences based on 1-way ANOVA followed by Tukey's HSD test (*P* < 0.05). Box plots were generated in BioRender (https://www.biorender.com).

Unlike in tobacco transgenics, the *T*_1_ seeds of *P_CL1_*-push + pull lines mostly failed to germinate, except for 2 individuals. Conversely, all *P_AtLHB1B1_*-push + pull lines germinated but they were hemizygous (Data S1), implicating that homozygosity of the *P_AtLHB1B1_*-push + pull transgene appears to impede germination. To assess metabolic changes that potentially impacted the seed germination, these different *T*_1_ seeds were subjected to metabolite analysis. While a slight red coloration was visible in EV seeds due to seed-specific RFP expression, the seeds of both push + pull lines showed strong red pigmentation (Fig. 5a). Indeed, betalain levels were elevated in both pull and push + pull seeds, though their levels did not significantly differ among them (Fig. 5b). Tyrosine levels were significantly higher in both push + pull seeds than in EV or pull seeds, with the *P_CL1_*-push + pull seeds accumulating significantly more tyrosine than the *P_AtLHB1B1_*-push + pull seeds (Fig. 5b). L-DOPA levels were likewise significantly elevated in both push + pull seeds relative to EV or pull seeds, although no significant difference was observed between the 2 push + pull genotypes (Fig. 5b). Phenylalanine levels were also significantly higher in both push + pull seeds compared with EV or pull seeds. Analysis of protein-bound amino acids showed that, while the levels of protein amino acids did not alter among genotypes overall, the level of protein-bound tyrosine significantly increased in *P_CL1_*-push + pull seeds compared with other genotypes (Fig. 5c and Figure S12). Therefore, the substantial accumulation of tyrosine in the seeds of *P_CL1_* but not *P_AtLHB1B1_*-push + pull lines, even in protein-bound pools, might have impacted its seed germination.

**Figure 6:**
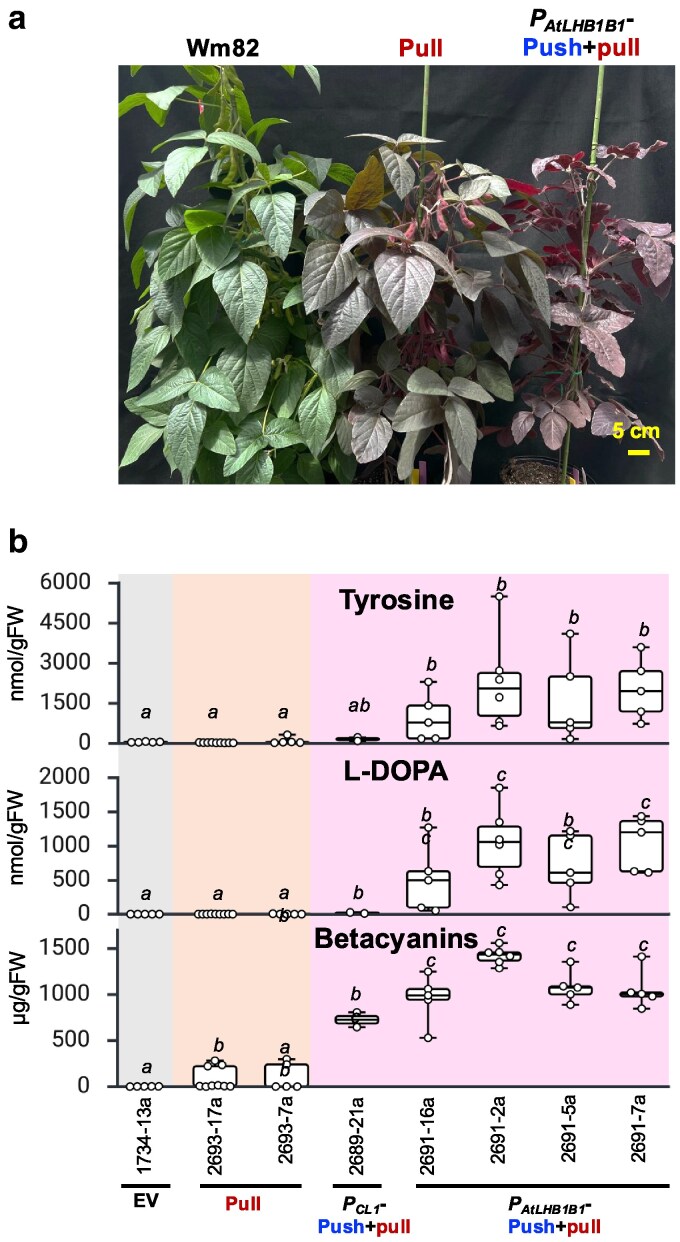
Leaves of *P_AtLHB1B1_*-push + pull lines show the strongest betalain pigmentation while finishing the whole life cycle in *T*_1_ generation. a) Phenotypes of stable *T*_1_ soybean lines of pull or push + pull constructs together with wild-type Williams 82 (Wm82). b) Quantification of tyrosine, L-DOPA, and betacyanin levels in mature leaves of soybean stable transgenic *T*_1_ lines. Tyrosine and L-DOPA levels were analyzed by LC–MS. Total betacyanin content was measured by absorbance at 538 nm using a spectrophotometer. The experiment was conducted using biological replicates, with sample sizes as follows: EV (*n* = 5), 2693-17a (*n* = 9), 2693-7a (*n* = 5), 2689-21a (*n* = 2), 2691-16a (*n* = 5), 2691-2a (*n* = 6), 2691-5a (*n* = 5), and 2691-7a (*n* = 5). Letters denote significant differences based on 1-way ANOVA of log_10_-transformed values followed by Tukey's HSD test (*P* < 0.05). Box plots were generated in BioRender (https://www.biorender.com).

Leaf metabolite analyses showed that the hemizygous *P_AtLHB1B1_*-push + pull *T*_1_ plants exhibited stronger pigmentation than the hemizygous pull lines without showing severe growth defects as seen in tobacco, except for slightly reduced seed yields and thinner leaves (Fig. 6a, Data S1). Consistent with their visual phenotype, the leaves of *P_AtLHB1B1_*-push + pull lines accumulated the highest betacyanin levels, followed by those of the *P_CL1_*-push + pull lines and pull-only lines (Fig. 6b). Targeted metabolite analysis further revealed elevated tyrosine and tyrosine-derived metabolites such as HGA, as well as increased levels of L-DOPA and its derivatives (e.g., dopamine) in *P_AtLHB1B1_*-push + pull lines. Conversely, phenylalanine and phenylpropanoids (e.g., *p*-coumarate) were reduced in *P_AtLHB1B1_*-push + pull lines, suggesting a redirection of metabolic flux toward tyrosine-derived pathways in these lines (Figure S13). Although such imbalances often lead to detrimental growth effects in other plant species (de Oliveira et al. 2019) including in tobacco (Fig. 4a), soybean *P_AtLHB1B1_*-push + pull lines largely tolerated these metabolic shifts while producing high levels of betalains (Fig. 6a).

## Discussion

### Betalain production can be scaled up using stable transgenic lines

Betalains are tyrosine-derived pigments, which are unique to the Caryophyllales in plants and are valued as natural food colorants due to their stability across a wide pH range (Calva-Estrada et al. 2022; Carreón-Hidalgo et al. 2022; Martins et al. 2024). Growing health and environmental concerns and regulatory pressures on petroleum-based synthetic dyes are accelerating the shift toward natural alternatives. However, beetroot, the primary source of commercial betalain production, is seasonally constrained and costly. Therefore, we need to establish a scalable and economically viable production platform of natural pigments (Akan et al. 2021; Novais et al. 2022; Thomsen et al. 2023; U.S. Food and Drug Administration, 2025a, 2025b). Betalain pathways have been successfully reconstructed using synthetic biology approaches in several plant species. Early stable transgenic work in Solanaceae generated modest pigment levels, 0.065 to 0.12 mg/g FW in eggplant, potato, and up to 0.25 mg/mL in tomato juice (Polturak et al. 2017). The polycistronic *RUBY* system (He et al. 2020) simplified pathway assembly and expanded betalain production to carrot (0.94 mg/g DW; Deng et al. 2023) and *N. benthamiana* (∼1.03 mg/g FW; Pramanik et al. 2024). Further pushing the precursor supply and debottlenecking the DODA step elevated *N. benthamiana* betacyanin yields to 2.87 mg/g FW (Jung and Maeda 2024), exceeding levels typically observed to accumulate in beet storage roots.

In this study, we advanced betalain engineering by demonstrating whole-plant, high-yield betalain production in stable transgenics by testing across 3 species—Arabidopsis, tobacco, and soybean. By combining the rearranged *RUBY* construct with an extra DODA enzyme (“pull”) and enhancing tyrosine precursor supply through BvTyrAα (“push”) and fine-tuning promoter strength (Fig. 1b), we achieved pigment levels that match or even exceed previous stable and transient systems. In tobacco, *P_AtLHB1B1_*-driven push + pull lines accumulated up to 7.31 mg/g FW (Fig. 2b), surpassing beetroot pigmentation (1.29 to 1.84 mg/g FW of betacyanins; Sawicki et al. 2016) and exceeding the highest previous betacyanin levels in *N. benthamiana* (2.87 mg/g FW; Jung and Maeda 2024). The use of the weaker *P_CL1_* promoter still maintained high yields (up to 4.38 mg/g FW) while mitigating growth defects (Fig. 2), underscoring the importance of balanced expression for sustained production.

Soybean push + pull lines displayed strong pigment accumulation across different tissues while maintaining robust growth and produced up to 1.28 and 1.56 mg/g FW betacyanins in *T*_0_ and *T*_1_ transgenic leaves, respectively (Fig. 3). The stable tobacco and soybean lines showed 2 major betacyanin species, betanin and isobetanin, like in beetroots (Sawicki et al. 2016; Xue et al. 2026), though our transgenics showed a higher proportion of betanin relative to isobetanin than beetroot (Figure S14). Recent studies demonstrated that the e*RUBY* construct expressing *BvTyrAα*, along with *CYP76AD1*, *DODA*, and *cDOPA5GT*, enabled betalain accumulation in cereals, producing 0.308 mg/g DW in rice endosperm (Tan et al. 2025) and 6.88 mg/g DW and 11.40 mg/g DW in maize kernels, depending on their background cultivar (Xue et al. 2026). These studies reinforce that balancing the enhanced tyrosine precursor supply *and* utilization is essential for achieving high betalain accumulation in stable transgenic plants. Our findings offer a framework to establish a sustainable and scalable production platform of betalains using stable transgenic plants.

### Transgene silencing and potential cytotoxicity hamper engineering efforts to obtain homozygous transgenic lines

Engineering metabolic pathways using multigene constructs in plants often encounters 2 major issues: posttranscriptional transgene silencing (PTGS) and metabolic cytotoxicity. A recent work reported that an unusual 3-consecutive-histidine motif within the *c*DOPA5GT coding sequence can cause ribosome stalling and activate No-Go RNA Decay, generating aberrant RNA fragments that feed into siRNA biogenesis and trigger PTGS (Kramer et al. 2025). In our study, the homozygous soybean “pull” lines behaved consistently with this mechanism. Their seedlings initially exhibited strong pigmentation but gradually lost betalains during vegetative development, ultimately showing patchy or nearly pigment-free leaves at maturity, whereas their hemizygous siblings maintained pigmentation throughout (Figure S11). This progressive loss of pigment in homozygous “pull” soybean lines closely resembles the “Red-to-Green” phenotype described by Kramer et al. (2025), in which *RUBY* expression diminishes due to PTGS. As Soybean “pull” lines lack elevated levels of tyrosine or L-DOPA (Fig. 6b, Data S1), metabolic toxicity is unlikely cause of their progressive loss of pigments. Instead, it is consistent with PTGS becoming more problematic as transgene dosage increases in the homozygous transgenics. To mitigate this issue, future construct designs should replace the *cDOPA5GT* gene with homologs from other species that lack the 3-histidine motif, which may help reduce ribosome stalling and improve pigment accumulation across developmental stages.

Conversely, the inability to recover homozygous soybean “push + pull” lines is likely attributed to metabolic cytotoxicity, where precursors or intermediates accumulate to certain levels that impose metabolic burden or cause damage to plant hosts. Soybean displayed greater tolerance to elevated tyrosine and L-DOPA than tobacco or Arabidopsis but still did not yield homozygous push + pull *T*_1_ plants—all recovered *T*_1_ were hemizygous (Data S1). The homozygous seeds failed to germinate, even before visible pigmentation occurred, suggesting that precursor/intermediate cytotoxicity, rather than silencing, likely led to lethality during embryogenesis or germination. Notably, other studies have successfully engineered betalain production in rice and maize seeds without reported adverse effects on seeds viability (Tan et al. 2025; Xue et al. 2026). This discrepancy may be derived from species-specific differences in metabolic tolerance, the timing and strength of transgene expression (eg, promoter strength, transgene dosage), or imbalances between precursor supply and utilization. Unlike the previous studies that used a polycistronic construct expressing all 4 genes under a single promoter (Tan et al. 2025; Xue et al. 2026), our study used separate promoters to express *BvTyrAα* and betalain genes. Therefore, a strong “push” activity might have driven excessive accumulation of upstream intermediates during embryogenesis or germination, before the “pull” capacity was sufficiently established in the downstream. Previous studies also observed that thiamine pathway engineering in Arabidopsis caused developmental abnormalities linked to toxic pyrimidine byproducts (Strobbe et al. 2021), while altering steroidal glycoalkaloid biosynthesis in tomato resulted in cholesterol overaccumulation and impaired development (Jozwiak et al. 2024). Therefore, metabolic flux must be carefully balanced to prevent cytotoxicity and to enable stable, heritable production of target metabolites.

Although hemizygous “pull” and “push + pull” soybean lines in this study maintained strong pigment production even in their *T*_1_ generation (Fig. 6, Data S1), obtaining homozygous lines would be desirable to eliminate segregation in future generations. Toward this next goal, we could use weaker, tissue-specific promoters (Zhang et al. 2015), or delay “push” expression to later developmental stages by using developmentally regulated promoters (eg, senescence-specific promoters; Gan and Amasino 1995). Enhancing “pull” capacity by using more active or compartmentalized “pull” enzymes may also help prevent early-stage lethal accumulation of intermediates (eg, L-DOPA). Additionally, compartmentalizing toxic intermediates, by targeting pathway enzymes and products to vacuoles, plastids, or synthetic storage structures, could further reduce potential toxicity of intermediates (Zhao et al. 2018). Therefore, future efforts to express transgenes at right time and location, while carefully tuning promoters and terminators (Diamos and Mason 2018), will likely enable the recovery of homozygous lines with high pigment yield.

### Promoter selection mitigates growth penalty while maintaining betalain production

Promoter choice plays a central role in shaping both metabolic flux and physiological tolerance in engineered plants (Huang et al. 2021; Jores et al. 2021; Yaschenko et al. 2022), particularly for pathways in which precursor imbalance or intermediate accumulation can impose substantial fitness costs. Despite this, most plant synthetic biology studies still rely on a narrow set of strong constitutive promoters, which maximizes expression but lacks fine control and increases the likelihood of recombination between repeated promoter sequences (Belcher et al. 2020; Zhou et al. 2023; Golubova et al. 2024). In this study, we systematically evaluated the impact of promoter strength on betalain production by expressing *BvTyrAα* under 2 promoters with distinct strengths, *P_AtLHB1B1_* and *P_CL1_*, to modulate tyrosine precursor supply across multiple plant species (Fig. 1b).

The *P_AtLHB1B1_* promoter consistently drove higher tyrosine accumulation and stronger pigmentation in leaves than the *P_CL1_* promoter, reflecting the higher expression levels driven by *P_AtLHB1B1_* (Figures S5, S6, S8, and S9); however, these elevated “push” activities also resulted in more severe growth defects, especially in tobacco (Fig. 2a). Although *P_AtLHB1B1_*-push + pull lines accumulated high betalain levels, they exhibited severe growth defects, including dwarfism, delayed development, and failure to complete life cycles in Arabidopsis and tobacco (Figures S2a and S10, Data S1). In contrast, *P_CL1_*-push + pull lines in all 3 species produced robust pigmentation while maintaining substantially healthier growth than their *P_AtLHB1B1_* counterparts. These results demonstrate that promoter strength directly influences the balance between pathway productivity and plant fitness. Therefore, fine-tuning promoter strength to avoid metabolic imbalance is essential to prevent precursor overload, toxic intermediate accumulation, and the physiological penalties.

**Figure 7:**
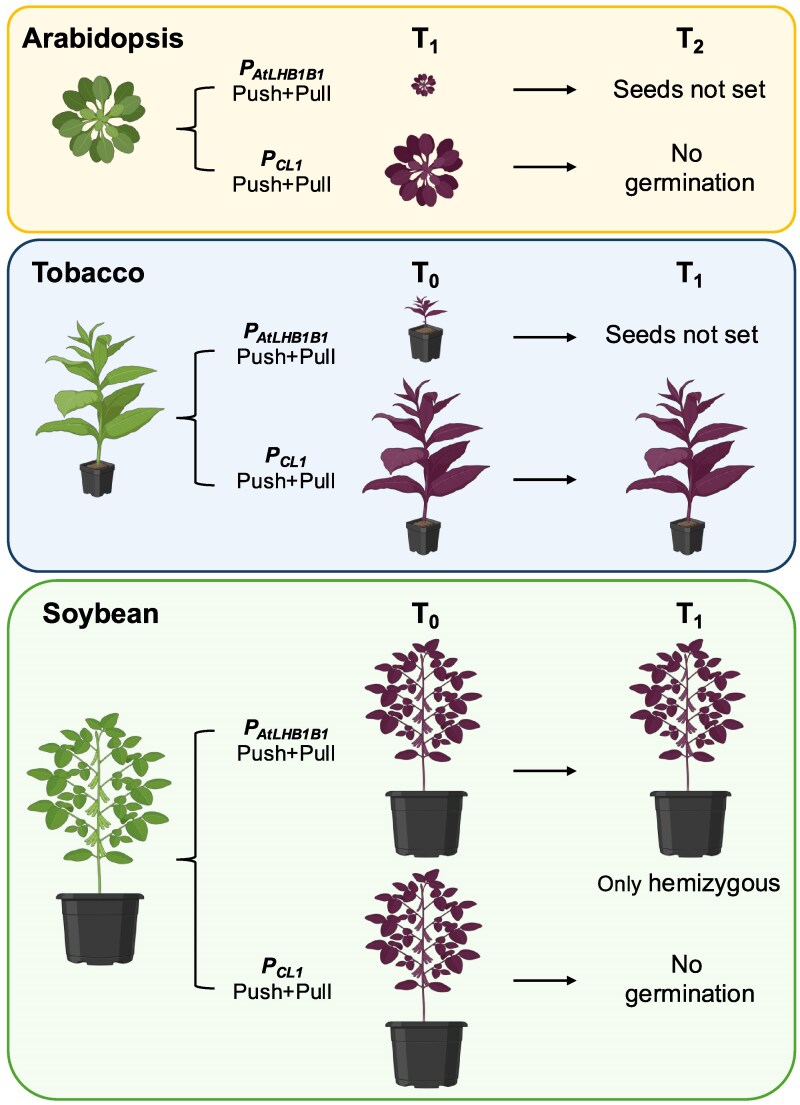
Cross-species comparison of betalain production reveals species- and/or promoter-dependent metabolic tolerance and life-cycle outcomes. Three distinct species (ie, Arabidopsis, tobacco, and soybean) were tested to stably express the optimized betalain pathway, combining an additional *DODA* and *RUBY*v2 (“pull”) with enhanced tyrosine precursor supply through BvTyrAα (“push”) expressed under either Arabidopsis light-harvesting chlorophyll–protein complex II subunit B1 (*P_AtLHB1B1_*) or Arabidopsis NADH dehydrogenase ubiquinone 1 beta subcomplex subunit promoter (*P_CL1_*). Arabidopsis, tobacco, and soybean images were generated in BioRender (https://www.biorender.com).

Importantly, our results also underscore that promoter selection must consider not only promoter strength but also spatiotemporal expression patterns. Although *P_CL1_*-push + pull lines of Arabidopsis and tobacco exhibited milder growth defects and successfully produced seeds, unlike their *P_AtLHB1B1_*-driven counterparts (Figure S10, Fig. 2a), none of the *P_CL1_*-push + pull seeds germinated in the next generation in Arabidopsis and soybean (Fig. 7, Data S1). Metabolite analysis of soybean *T*_1_ seeds further showed that *P_CL1_*-push + pull seeds accumulated substantially higher levels of both free and protein-bound tyrosine than other lines (Fig. 5). This metabolic alteration likely reflects unintended high expression of *P_CL1_* in germinating seeds, especially in early developmental stages (Klepikova et al. 2016), which may have increased tyrosine levels during embryogenesis or early germination, ultimately impairing seed viability. Thus, promoters with desirable average strength may still impose unintended developmental issues if their activity is not spatially and developmentally restricted.

These results highlight that promoter choice should be treated as a core design decision during the Design phase of the DBTL cycle of plant synthetic biology. Recent technological advances including constitutive promoter libraries with graded strengths (Zhou et al. 2023), synthetic promoters with tunable output (Cai et al. 2023), transcriptional repression toolkits (Markel et al. 2024), and synthetic regulatory circuits enabling spatial patterning or logic-based control (Brophy et al. 2022) offer powerful opportunities to regulate biosynthetic gene expression more precisely. Given the intricate spatial and temporal complexity of plant specialized metabolism (Sweetlove and Fernie 2013), integrating such regulatory tools into pathway engineering will enable more balanced flux distribution, reduced metabolic burden, and enhanced stability and scalability of target compound production in plants. Overall, our findings demonstrate that strategic promoter selection is essential for maximizing betalain yields while minimizing unintended growth defects.

### Species-dependent differences in metabolic tolerance against synthetic engineered pathways underscore the importance of chassis selection

Different species vary widely in their background metabolism (eg, precursor availability) and their responses to the introduction of an engineered pathway. Although primary metabolism is largely conserved across the plant kingdom, species-specific differences in precursor biosynthesis, feedback regulation, and metabolic compartmentalization can influence the efficiency of heterologous chemical production (Maeda 2019; Maeda and Fernie 2021; Selma et al. 2023). Consistent with this notion, our cross-species comparison revealed substantial variations in tolerance to the engineered betalain pathway (Fig. 7). Arabidopsis exhibited the lowest tolerance, showing severe dwarfism, sterility, or germination failure in “push + pull” lines (Figure S10). Tobacco displayed moderate tolerance, supporting high betalain production in *T*_0_ push + pull lines, yet experiencing promoter-dependent growth defects, particularly under strong *P_AtLHB1B1_*-driven “push + pull” expression (Fig. 2). In contrast, soybean exhibited the highest tolerance, among 3 plant species, to the introduced betalain pathway (Fig. 7). Soybean push + pull lines accumulated high levels of tyrosine, L-DOPA, and downstream derivatives, yet maintained robust vegetative growth, completed life cycles, and produced viable hemizygous *T*_1_ progeny with strong pigmentation (Figs 3 and 6).

This high tolerance in soybean may be due to its dual-tyrosine biosynthetic pathways via the canonical plastidial route along with the cytosolic pathway mediated by a feedback-insensitive TyrA prephenate dehydrogenase (Schenck et al. 2015, 2017). This unique and flexible pathway configuration in legumes likely enhances tolerance to elevated levels of tyrosine precursor, pathway intermediates, and their derivatives. Species-dependent effects have been observed upon metabolic engineering of other pathways, such as vanillin production, where rice and pepper showed differences in ferulic acid precursor availability and tolerance (Chee et al. 2017; Arya et al. 2022). Together, the endogenous metabolic background of host plants strongly influences the performance of an introduced engineered pathway.

This finding emphasizes that chassis selection must be viewed as a critical design parameter; just because a plant is amenable to an easy transformation method (eg, floral dipping for Arabidopsis, leaf Agro-infiltration for *N*. *benthamiana*) should not be the main driver of achieving ultimate goals of efficient chemical production. The underpinning biochemistry and physiology of the host plants may not effectively support the engineering of a target pathway. Screening of candidate plant species for unique endogenous metabolism and precursor availability can help identify promising hosts with greater intrinsic metabolic tolerance, thereby reducing the risk of developmental defects and improving overall product yield. Ultimately, expanding plant synthetic biology beyond *N. benthamiana* to include diverse, stably transformable crops will be crucial for leveraging host-specific metabolic compatibilities and enabling efficient, large-scale biosynthesis of valuable specialized metabolites.

### Conclusions

Plant synthetic biology provides an exciting opportunity to harness and reprogram plant metabolism for sustainable and scalable production of high-value natural compounds (Liu and Stewart 2015; Zhu et al. 2021). Engineered soybean plants generated in this study offer a potential carbon-neutral platform to produce natural pigments, by harnessing sunlight energy and converting atmospheric CO_2_ into valuable compounds without reliance on fossil fuels. As soybean is a widely cultivated commodity crop, betalain pigment extraction from red soybeans could occur alongside conventional soybean processing pipelines. Such dual-use value streams could greatly enhance the economic feasibility of plant-based pigment production to meet the rapidly increasing need for safe, natural pigment alternatives to synthetic food dyes. As plant synthetic biology continues to advance, future progress will depend on integrating tunable promoters, optimal metabolic regulation, and careful chassis selection with rigorous assessment of metabolic impacts on both endogenous and engineered pathways. Expanding these design principles across diverse, high-biomass crop species will be critical for establishing reliable, climate-aligned, and commercially scalable production systems for betalains and other high-value compounds using plants as sustainable chemical production platforms.

## Materials and methods

### Generation of recombinant DNA constructs

All recombinant DNAs were generated using the Golden Gate (GG) cloning method (Engler et al. 2008, 2014; Weber et al. 2011; Werner et al. 2012; Patron et al. 2015). The intron insertion site within the BvTyrAα coding sequence was predicted using the NetGene2 server (https://services.healthtech.dtu.dk/services/NetGene2-2.42/). The full BvTyrAα coding sequence containing the potato ST-LS1 intron sequence (Eckes et al. 1986; Vancanneyt et al. 1990; Brophy et al. 2022) was synthesized in pUC57-Kan vector (Synbio Technologies, NJ, United States).

The rearranged *RUBY* polycistron, named in this work as *RUBY*v2, was generated using the RIGGERv2 system within the HARBOR system (manuscript in preparation) designed and used for GG-mediated plasmid assembly in the Molecular Technologies Department within the Wisconsin Crop Innovation Center (WCIC) at the University of Wisconsin—Madison. Briefly, genes included within the tri-cistronic *RUBY*v2 were integrated as CDS1ns Level 0 parts, into RIGGER receiver plasmids which have partial 2A peptide sequences on their termini. Due to RIGGERv2 system design constraints all incoming synthetic CDS1ns molecules (GenScript, Piscataway, NJ, United States; kanamycin resistance backbones) were first liberated from their subtending plasmids via restriction digestion with *Bsa*IHFv2 (New England Biolabs (NEB), Ipswich, MA, United States) followed by SB buffered agarose gel-mediated size separation and purification of the linear fragments. Specifically, the 828 base pair (bp) *DODA* gene (from plasmid GS-0529) was installed, via T4 DNA ligase–mediated ligation, in the *Bbs*IHF (NEB) digested RIGGER F2A Position 1 receiver (plasmid stock number RC5821-RCGG5030, chloramphenicol resistance backbone; 1,781 bp fragment purified away from released lacZ promoter driven β-galactosidase scorable marker gene, which occupies the cloning interval in all unmodified RIGGER receivers); the Postion 1 DODA plasmid was named RC7279A-RCGG7044. The 1,503 bp *c*DOPA5GT fragment (from plasmid GS-0530) was installed, in a *Bbs*IHF (NEB)-mediated GG reaction, in the RIGGER F2A Position 2 receiver (plasmid stock number RC5822-RCGG5031, carbenicillin (ampicillin) resistance backbone); the reaction also included the RIGGER T2A triscistronic module (plasmid stock number RC5818-RCGG5027, gentamicin resistance backbone) which provides the ability to connect a downstream gene in the subsequent multifragment assembly (detailed below); the completed *c*DOPA5GT in the Position 2 receiver was named RC7280A-RCGG7045. The 1,494 bp CYP76AD1 fragment from plasmid GS-0528 was ligated with *Bbs*IHF digested, gel purified 1,966 bp backbone of the RIGGER T2A Position 3 receiver (RC5823-RCGG5032, carbenicillin (ampicillin) resistance backbone) and the *Bbs*IHF digested, gel purified 963 bp fragment, containing the L-rhamnose inducible promoter driven monomeric Red Fluorescent Protein, of the RIGGER STOP module (plasmid stock number RC5820-RCGG5029, gentamicin resistance backbone). *Escherichia coli* colonies containing the successfully assembled CYP76AD1 fragment in the RIGGER T2A Position 3 receiver (and with the RIGGER STOP) exhibited a red color when cultured on solid lysogeny broth (LB) medium supplemented with carbenicillin and 100 mM l-rhamnose; the completed CYP76AD1 plasmid was named RC7281A-RCGG7046. The aforementioned RIGGER receiver plasmids which had been modified with the DODA, *c*DOPA5GT, and CYP76AD1 molecules were then combined in an *Esp3*I (NEB)-mediated multifragment GG reaction. The resulting *RUBY*v2 polycistronic Level 0 (L0) part in plasmid RC7282A-RCGG7047, which produces a red colored colony on solid LB supplemented with chloramphenicol and 100 mM l-rhamnose, features a pair of convergently oriented *Bsa*I recognition sequences that produce the AATG–GCTT overhangs necessary for successful integration of the *RUBY*v2 CDS1 in transcriptional units produced in Level 1 (L1) GG reactions in concordance with standard MoClo system GG reactions that were subsequently executed to build the transcriptional units described below.

Since the *P_CL1_* promoter contained an internal *Bbs*I restriction site, the promoter was amplified as 2 separate fragments to domesticate the internal *Bbs*I site using primers listed in Table S1. The resulting domesticated promoter, flanked with *Bbs*I sites and overhangs compatible with the pAGM1251 were assembled into the L0 acceptor pAGM1251. All other L0 parts were obtained from either the MoClo toolkit (Engler et al. 2014) or Jung and Maeda (2024). These L0 parts were assembled into L1 acceptors using the Golden Gate reaction with promoter, C-terminal tag, and terminator parts as described in Data S4. The generated L1 parts were further used for Level 2 (L2) assembly to generate EV, “push,” “pull,” and “push + pull” constructs as described in Data S4 and used for transient expression assay, floral dipping, and tissue culture.

Binary plasmid constructs were assembled using the WCIC AtdTP-SPEC BASE plasmid (RC4836-RCGG3779, also the Maeda lab stock number bHM1450). This RK2 replicon contains a constitutively expressed *aadA1a* (Hollingshead and Vapnek 1985; synthetic L0 CDS1 part, codon optimized for use in soybean, stock number S018030-7), which confers to transgenic plant cells the ability to resist spectinomycin selection. Additionally, the *aadA1a* is fused with the At2g04842 bi-organellar targeting sequence (Berglund et al. 2009; synthetic L0 NT1 part stock number S018030-5), which was previously shown to direct fused proteins to both plastids and mitochondria; the use of the AtdTP as a targeting signal for aadA1a results in increased transformation efficiency for multiple plant taxa and is the core of a recent WCIC patent application (Petersen et al. 2022). Expression of the *AtdTP-aadA1a* is controlled by the 2× enhanced Cauliflower Mosaic Virus (CaMV) 35S promoter (L0 Pro part, pICH45089; Odell et al. 1985; Kay et al. 1987), in conjunction with the Tobacco Mosaic Virus Ω translational enhancer (L0 NT1 part, pAGT707; Gallie et al. 1987); polyadenylation of the spectinomycin resistance gene is controlled by the CaMV 35S terminator (Hirt et al. 1990; Irniger et al. 1992; L0 3 U + TER part, stock number pICH41414). The 2× enhanced CaMV35S promoter TMV Ω driven *AtdTP-aadA1a* CaMV35S terminator transcriptional unit (TU) was assembled in the MoClo pL1R-1 L1 acceptor (plasmid stock number pICH47802), which results in the unit being adjacent to, with transcriptional progress directed toward, the T-DNA Left Border sequence; the completed L1 plasmid stock number is RC4025-RCGG2915. The WCIC AtdTP-SPEC BASE was completed by installation of the aforementioned L1 TU, along with the MoClo pELB-2 unit (which features a pair of divergently oriented *Bsa*I sites flanking the lacZ promoter driven β-galactosidase scorable marker gene; this allows for subsequent modification of the L2 plasmid) in the MoClo RK2 base L2 plasmid (stock number pAGM4673) in a *Bbs*IHF-mediated GG reaction to produce RC4836-RCGG3779. The sequences of final L2 constructs were verified by whole-plasmid sequencing (Plasmidsaurus, KY, United States).

### Transient expression of generated DNA constructs in *N. benthamiana*

Transient expression of recombinant DNA constructs in *N. benthamiana* leaves was conducted following Jung and Maeda (2024). *N. benthamiana* plants were grown under ∼200 *μ*E of light intensity with a 12 h/12 h (light/dark) at 24 °C and 60% humidity. The generated binary vectors were transformed into *Agrobacterium tumefaciens* strain GV3101::pMP90 (Koncz and Schell 1986) using electroporation (McCormac et al. 1998). *A. tumefaciens* strains harboring each construct were grown in 10 mL of LB (Bertani 1951, 2004) with antibiotics at 28 °C in a shaking incubator (New Brunswick Innova 40R) at 250 rpm for ∼24 h. Overnight saturated cultures were centrifuged at 3,000 × *g* for 5 min at room temperature. The pellets were washed twice with 3 mL of induction media [10 mm 2-(N-morpholine)-ethanesulphonic acid (MES) pH 5.6, 0.5% (w/v) glucose, 2 mm NaH_2_PO_4_, 20 mm NH_4_Cl, 1 mm MgSO_4_, 2 mm KCl, 0.1 mm CaCl_2_, 0.01 mm FeSO_4_, and 0.2 mm acetosyringone] and incubated in the induction medium for 2 to 3 h at room temperature in dark. After the incubation, the cells were pelleted at 3,000 × *g* for 5 min at room temperature and resuspended in 3 mL of 10 mm MES pH 5.6 with 0.2 mm acetosyringone. All resuspended cultures were diluted to OD_600nm_ of 0.5 for infiltration. Four-week-old plants were infiltrated on the abaxial side of the leaf using a 1 mL needleless syringe (Becton, Dickinson and Company, 309659) and grown for 3 d under the same condition as described above before sample collection.

### Generation of stable transgenic lines

After sequencing the level 2 binary vectors, plasmids were transformed into *A. tumefaciens* GV3101::pMP90 by electroporation. Colonies were confirmed by PCR and then stored in −80 °C until further use. Stable transgenic Arabidopsis lines were generated using a modified floral dip method (Yokoyama et al. 2021) with 5- to 6-wk-old Arabidopsis Col-0 plants. The plasmids were also used to produce clones of *Agrobacterium rhizogenes* strain 18r12v (Collier and Taylor, unpublished) for use in the dicot meristem soybean transformation protocol used for generation of transgenic Williams 82 cultivar soybean lines at the WCIC.

Stable transgenic lines of tobacco were generated following Yau et al. (2020) with minor modifications. Seeds of transgenic tobacco (*N. tabacum* L. cultivar “Petit Havana” SR1) were sterilized with 70% (v/v) ethanol for 2 min, bleached with 30% (v/v) sodium hypochlorite and drops of Triton X-100 for 20 min and washed thoroughly with autoclaved distilled water. Sterilized seeds were germinated on germination medium (MS medium with vitamin [Research Products International, Cat. No. M10400], 3% [w/v] sucrose, 0.8% [w/v] agar, pH 5.8). Plates were sealed with medical air-permeable tape and placed in a 25 °C growth chamber with 12 h/12 h (light/dark) photoperiod for 2 wk. The day before inoculation, Agrobacterium strains were streaked on LB plates containing antibiotics and allowed to grow at 30 °C for 1 d. Agrobacterium colonies were suspended in transformation medium (MS medium with vitamin [Research Products International, Cat. No. M10400], 3% [w/v] sucrose, pH 5.8, 3 *μ*g/mL 6-benzylaminopurine [PhytoTechnology Laboratories, Product No. I885], and 100 *μ*m acetosyringone [Thermo Scientific, Cat. No. 115540010]) and were then diluted 10 times with the same medium for genetic transformation. Cotyledons of 2-wk-old seedlings were gently bruised with a sterilized toothpick dipped into the desired Agrobacterium suspension. After inoculation, the cotyledons were placed abaxial on co-cultivation medium (transformation medium + 0.8% agar) for 3 d in the dark and then transferred to selection medium (transformation medium + 0.8% agar + carbenicillin [500 *μ*g/mL] + spectinomycin [50 *μ*g/mL]) with the leftover stem sticking into the medium. Plates were sealed with medical air-permeable tape and placed in a 25 °C growth chamber with 12 h/12 h (light/dark) photoperiod. Subculturing was carried out every 2 wk. After shoots were regenerated, shoots were excised from the callus and transferred into rooting medium, MS medium with vitamin (Research Products International, Cat. No. M10400), 3% (w/v) sucrose, 0.8% agar, 1-naphthaleneacetic acid (1 *μ*g/mL), + carbenicillin (500 *μ*g/mL) + Spectinomycin (50 *μ*g/mL). After roots were regenerated, each transgenic plant was carefully moved to soil and grown in a 25 °C growth chamber with 12 h/12 h (light/dark) photoperiod.

### Metabolite extraction and analysis

To measure metabolite levels, leaf discs were harvested, flash-frozen in liquid nitrogen, ground to powder, and kept at −80 °C until use. Approximately 20 to 30 mg of frozen powder was mixed in 400 *μ*L of the extraction buffer containing 2:1 (v/v) methanol:chloroform including ^13^C ring-labeled phenylalanine as an internal standard. After adding 300 *μ*L of H_2_O and then 125 *μ*L of chloroform, samples were centrifuged at 10,000 × *g* for 5 min. Each 225 *μ*L of the polar phase was transferred to 2 fresh tubes, dried down overnight in a SpeedVac at room temperature, and resuspended in either 100 *μ*L of LC–MS grade water or 100 *μ*L of 50% (v/v) methanol before spectrophotometry or LC–MS analysis, respectively.

For LC–MS analysis, 1 μL of each sample resuspended with 50% (v/v) methanol was injected onto a HSS T3 C18 reversed phase column (100 × 2.1 mm i.d., 1.8 *μ*m particle size; Waters, Milford, United States) and eluted using a 27-min gradient comprising 0.1% (v/v) formic acid in LC–MS grade water (Solvent A) and 0.1% (v/v) formic acid in 90% (v/v) LC–MS-grade acetonitrile (Solvent B) at a flow rate of 0.4 mL/min and column temperature of 40 °C. The binary linear gradient with the following ratios of solvent B was used: 0 to 1 min, 1%; 1 to 10 min, 1% to 10%; 10 to 13 min, 10% to 25%; 13 to 18 min, 25% to 99%; 18 to 22 min, 99%; 22% to 23.5 min, 99% to 1%; 23.5 to 27 min, 1%. The MS spectra were recorded using the full scan in positive mode, under the following parameters: mass range, 100 to 1,500 mass/charge ratio (*m*/*z*); resolution, 70,000; maximum scan time, 100 ms; AGC target, 1 × 10^6^; capillary temperature, 350 °C; heater temperature, 150 °C; spray voltage, 3.5 kV. For MS2, the MS spectra were recorded under the following parameters: resolution, 17,500; maximum scan time, 50 ms; minimum AGC target, 8 × 10^3^; AGC target, 2 × 10^5^; collision energies, normalized to *m/z* 500, *z* = 1; apex trigger: 1 to 180 s. The identity of each metabolite peak was confirmed by comparing their accurate masses and retention times with those of the corresponding authentic standards. Quantification was based on the standard curves generated by injecting different concentrations of authentic chemical standards.

Untargeted metabolite analysis was conducted following (El-Azaz and Maeda 2024), with minor modifications. High-throughput integration was conducted in MZmine v4.1.0 (Schmid et al. 2023) using full-range TIC (*m/z* 100 to 1,500) positive ionization data collected between 1 and 27 min. For feature detection, the noise threshold was set to 1.0 × 10^4^ and 2.0 × 10^3^ for MS1 and MS2, respectively. Chromatograms were built with the LC–MS chromatogram builder tool for mass features with a minimum absolute height of ≥2.0 × 10^5^ and detected in at least 5 consecutive scans, with a minimum intensity of 1.0 × 10^6^ between peaks. Local minimum resolver was applied for a minimum ratio of peak top/edge of 3 and a maximum peak duration of 1 min. Carbon-13 isotopes were then removed using the ^13^C isotope filter tool. Features were aligned with the join aligner tool using a retention time and *m*/*z* tolerance of 0.2 min and 10 ppm, respectively. Redundant features were consolidated using the duplicate feature filter. Features present in at least 3 out of all individuals within the same genotype were subtracted from the feature list, except for features at least 3 times more abundant in the plant samples compared with the blank, which were kept. Gaps in the blank-subtracted feature list were filled using the feature finder tool. After gap-filling, features not present in at least 5 plant samples were removed using the list rows filter tool, and a correlation analysis of the remaining features was performed in metaCorrelate. The final feature list was then exported as both molecular networking files and SIRIUS outputs with merged MS2 spectra. The exported feature list was further analyzed in MS excel, the integrated peak area divided by the mass of the plant sample (in g FW), and the recovery factor of ^13^C ring-labeled phenylalanine (determined in a manual integration) to perform statistical analysis across the dataset. The SIRIUS output file was used to predict feature identity and structure in SIRIUS v5.8.6 (Dührkop et al. 2019), allowing [M + H^+^], [M + Na^+^] and [M + K^+^] as possible ionizations. ZODIAC (Ludwig et al. 2020) was enabled at default parameters to improve the search. A structure search was performed with the CSI:FingerID (Dührkop et al. 2015) in all available databases.

Betacyanin quantification was conducted following Jung and Maeda (2024). Briefly, 30 *μ*L of sample resuspended in LC–MS grade water were transferred to individual wells of a 96 Greiner transparent plate, flat bottom, half well size (#675101) with 5 times serial dilutions. Absorbance at 538 nm was measured using a plate reader (Infinite 200 PRO, TECAN). Multiple data points in the linear range were used for further analysis. Absorbance values were converted to betacyanin content using the molar extinction coefficient *ε* = 60,000 M^−1^ cm^−1^ (Stintzing et al. 2003).

### Amino acid analysis

Protein-bound amino acid was performed as described in (Yobi and Angelovici 2018). Briefly, ∼3 mg dry seeds (*n* = 6) were hydrolyzed with 6N HCl for 24 h at 110 °C. After hydrolysis and filtration, 10 *µ*L of the hydrolysate was transferred to clean tubes and evaporated to dryness. The pellets were then reconstituted with Milli-Q purified water containing 13 internal standards and analyzed using a Xevo TQ Absolute ultra-performance liquid chromatography-tandem mass spectrometer instrument, UPLC–MS/MS (Waters Corporation, Milford, MA, United States) as described in Yobi and Angelovici (2018). During acid hydrolysis, tryptophan and cysteine were lost, asparagine was converted to aspartic acid, and glutamine was converted to glutamic acid; therefore, Asx represents a combination of asparagine and aspartate, and Glx represents a combination of glutamine and glutamate. This treatment also destroys all the free amino acids, leaving only the protein-bound amino acids. Serial dilutions of external standards were analyzed alongside the samples for accurate identification and quantification. Separation was done with a Kinetex LC column (2.6 *µ*m, C18, 100 Å, 100 × 21 mm; Phenomenex, Torrance, CA, United States) maintained at 30 °C. The injection volume was set to 10 *µ*L and the flow rate was set to 0.3 mL/min. The mobile phase A consisted of 1 mm of the ion-pairing agent perfluoroheptanoic acid, while acetonitrile served as the mobile phase B. The flow gradient was set as follows for B: 98% at 0 min; 80% at 0.1 min; 60% at 2.3 to 3.6 min; 98% at 4.0 to 5.98 min. The MS electrospray ionization in positive mode and multiple reaction monitoring transition for each compound were used for acquisition of mass spectra. Flow gas and desolvation were set to 150 and 500 L/h, respectively. Desolvation temperature was set to 350 °C, and the collision gas flow was set to 0.15 mL/min. The data were retrieved and analyzed using the MassLynx data analysis software (TargetLynx XS, Waters, Inc.), exported to Excel sheets and back calculated to total volume and sample weight to obtain the final amounts in nmol/mg tissue.

### RT-qPCR expression analysis

To test the expression levels of transgenes in the stable transgenic lines, transcript levels of transgenes were analyzed by reverse-transcription quantitative PCR (RT-qPCR) from the same frozen tissues used for corresponding metabolite analyses. Total RNA was isolated from ∼50 to 100 mg of frozen ground tissue using TRIzol (Invitrogen), treated with deoxyribonuclease I (Thermo Fisher Scientific) and reverse-transcribed to synthesize cDNA with M-MLV reverse transcriptase and random hexamer primers (Promega) according to the manufacturer's protocol. qPCR was conducted in a Stratagene Mx3000P (Agilent Technologies) thermocycler using GoTaq qPCR Master Mix (Promega). Specific primers used for the target genes are listed in Table S1. Expression of the *NtPP2A* gene and *GmUbi3* gene were used to normalize the sample-to-sample variations between different cDNA preparations in *N. tabacum* and *G. max*, respectively (Schmidt and Delaney 2010; Beyer et al. 2021). Relative expression levels among different infiltrated leaves were analyzed for each transgene using the 2^−ΔΔCt^ method (Livak and Schmittgen 2001).

## Supplementary Material

kiag337_Supplementary_Data

## Data Availability

The data underlying this article are available in the article and in its online supplementary material.

## References

[kiag337-B1] Akan S, Tuna Gunes N, Erkan M. 2021. Red beetroot: health benefits, production techniques, and quality maintaining for food industry. J Food Process Preserv. 45:e15781. 10.1111/jfpp.15781.

[kiag337-B2] Arya SS et al 2022. Metabolic engineering of rice cells with vanillin synthase gene (VpVAN) to produce vanillin. Mol Biotechnol. 64:861–872. 10.1007/s12033-022-00470-8. 35192168

[kiag337-B3] Barnum CR, Endelman BJ, Shih PM. 2021. Utilizing plant synthetic biology to improve human health and wellness. Front Plant Sci. 12:691462. 10.3389/fpls.2021.691462. 34504505 PMC8421571

[kiag337-B4] Belcher MS et al 2020. Design of orthogonal regulatory systems for modulating gene expression in plants. Nat Chem Biol. 16:857–865. 10.1038/s41589-020-0547-4. 32424304

[kiag337-B5] Berglund A-K et al 2009. Dual targeting to mitochondria and chloroplasts: characterization of Thr–tRNA synthetase targeting peptide. Mol Plant. 2:1298–1309. 10.1093/mp/ssp048. 19995731

[kiag337-B6] Bertani G . 1951. Studies on lysogenesis. I. The mode of phage liberation by lysogenic *Escherichia coli*. J Bacteriol. 62:293–300. 10.1128/jb.62.3.293-300.1951. 14888646 PMC386127

[kiag337-B7] Bertani G . 2004. Lysogeny at mid-twentieth century: P1, P2, and other experimental systems. J Bacteriol. 186:595–600. 10.1128/JB.186.3.595-600.2004. 14729683 PMC321500

[kiag337-B8] Beyer SF et al 2021. Disclosure of salicylic acid and jasmonic acid-responsive genes provides a molecular tool for deciphering stress responses in soybean. Sci Rep. 11:20600. 10.1038/s41598-021-00209-6. 34663865 PMC8523552

[kiag337-B9] Brophy JAN et al 2022. Synthetic genetic circuits as a means of reprogramming plant roots. Science. 377:747–751. 10.1126/science.abo4326. 35951698

[kiag337-B10] Cai Y-M, Witham S, Patron NJ. 2023. Tuning plant promoters using a simple split luciferase method to assess transcription factor-DNA interactions. ACS Synth Biol. 12:3482–3486. 10.1021/acssynbio.3c00094. 37856867 PMC10661027

[kiag337-B11] Calva-Estrada SJ, Jiménez-Fernández M, Lugo-Cervantes E. 2022. Betalains and their applications in food: the current state of processing, stability and future opportunities in the industry. Food Chem Mol Sci. 4:100089. 10.1016/j.fochms.2022.100089. PMC8991513 35415668

[kiag337-B12] Carreón-Hidalgo JP, Franco-Vásquez DC, Gómez-Linton DR, Pérez-Flores LJ. 2022. Betalain plant sources, biosynthesis, extraction, stability enhancement methods, bioactivity, and applications. Food Res Int. 151:110821. 10.1016/j.foodres.2021.110821. 34980373

[kiag337-B13] Chee MJY, Lycett GW, Khoo T-J, Chin CF. 2017. Bioengineering of the plant culture of *Capsicum frutescens* with vanillin synthase gene for the production of vanillin. Mol Biotechnol. 59:1–8. 10.1007/s12033-016-9986-2. 27826796

[kiag337-B14] Collier R et al 2017. Accurate measurement of transgene copy number in crop plants using droplet digital PCR. Plant J. 90:1014–1025. 10.1111/tpj.13517. 28231382

[kiag337-B15] de Felipe P et al 2006. *E unum pluribus*: multiple proteins from a self-processing polyprotein. Trends Biotechnol. 24:68–75. 10.1016/j.tibtech.2005.12.006. 16380176

[kiag337-B16] de Oliveira MVV et al 2019. Imbalance of tyrosine by modulating TyrA arogenate dehydrogenases impacts growth and development of *Arabidopsis thaliana*. Plant J. 97:901–922. 10.1111/tpj.14169. 30457178

[kiag337-B17] Deng Y-J et al 2023. Generating colorful carrot germplasm through metabolic engineering of betalains pigments. Hortic Res. 10:uhad024. 10.1093/hr/uhad024. 37786858 PMC10541523

[kiag337-B18] Diamos AG, Mason HS. 2018. Chimeric 3′ flanking regions strongly enhance gene expression in plants. Plant Biotechnol J. 16:1971–1982. 10.1111/pbi.12931. 29637682 PMC6230951

[kiag337-B19] Dührkop K et al 2019. SIRIUS 4: a rapid tool for turning tandem mass spectra into metabolite structure information. Nat Methods. 16:299–302. 10.1038/s41592-019-0344-8. 30886413

[kiag337-B20] Dünser K et al 2022. Endocytic trafficking promotes vacuolar enlargements for fast cell expansion rates in plants. eLife. 11:e75945. 10.7554/eLife.75945. 35686734 PMC9187339

[kiag337-B21] Dührkop K, Shen H, Meusel M, Rousu J, Böcker S. 2015. Searching molecular structure databases with tandem mass spectra using CSI:FingerID. Proc Natl Acad Sci U S A. 112:12580–12585. 10.1073/pnas.1509788112. 26392543 PMC4611636

[kiag337-B22] Eckes P, Rosahl S, Schell J, Willmitzer L. 1986. Isolation and characterization of a light-inducible, organ-specific gene from potato and analysis of its expression after tagging and transfer into tobacco and potato shoots. Mol Gen Genet. 205:14–22. 10.1007/BF02428027.

[kiag337-B23] El-Azaz J, Maeda HA. 2024. A simplified liquid chromatography-mass spectrometry methodology to probe the shikimate and aromatic amino acid biosynthetic pathways in plants. Plant J. 120:2286–2304. 10.1111/tpj.17105. 39466904 PMC11629745

[kiag337-B24] Engler C et al 2014. A golden gate modular cloning toolbox for plants. ACS Synth Biol. 3:839–843. 10.1021/sb4001504. 24933124

[kiag337-B25] Engler C, Kandzia R, Marillonnet S. 2008. A one pot, one step, precision cloning method with high throughput capability. PLoS One. 3:e3647. 10.1371/journal.pone.0003647. 18985154 PMC2574415

[kiag337-B26] Gallie DR, Sleat DE, Watts JW, Turner PC, Wilson TMA. 1987. The 5′-leader sequence of tobacco mosaic virus RNA enhances the expression of foreign gene transcripts in vitro and in vivo. Nucleic Acids Res. 15:3257–3273. 10.1093/nar/15.8.3257. 3575095 PMC340728

[kiag337-B27] Gan S, Amasino RM. 1995. Inhibition of leaf senescence by autoregulated production of cytokinin. Science. 270:1986–1988. 10.1126/science.270.5244.1986. 8592746

[kiag337-B28] Gandía-Herrero F, Escribano J, García-Carmona F. 2016. Biological activities of plant pigments betalains. Crit Rev Food Sci Nutr. 56:937–945. 10.1080/10408398.2012.740103. 25118005

[kiag337-B29] Golubova D, Tansley C, Su H, Patron NJ. 2024. Engineering *Nicotiana benthamiana* as a platform for natural product biosynthesis. Curr Opin Plant Biol. 81:102611. 10.1016/j.pbi.2024.102611. 39098308

[kiag337-B30] Grützner R et al 2021. Engineering betalain biosynthesis in tomato for high level betanin production in fruits. Front Plant Sci. 12:682443. 10.3389/fpls.2021.682443. 34177999 PMC8220147

[kiag337-B31] He Y, Zhang T, Sun H, Zhan H, Zhao Y. 2020. A reporter for noninvasively monitoring gene expression and plant transformation. Hortic Res. 7:152. 10.1038/s41438-020-00390-1. 33024566 PMC7502077

[kiag337-B32] Hirt H, Kögl M, Murbacher T, Heberle-Bors E. 1990. Evolutionary conservation of transcriptional machinery between yeast and plants as shown by the efficient expression from the CaMV 35S promoter and 35S terminator. Curr Genet. 17:473–479. 10.1007/BF00313074. 2202523

[kiag337-B33] Hollingshead S, Vapnek D. 1985. Nucleotide sequence analysis of a gene encoding a streptomycin/spectinomycin adenyltransferase. Plasmid. 13:17–30. 10.1016/0147-619X(85)90052-6. 2986186

[kiag337-B34] Huang D, Kosentka PZ, Liu W. 2021. Synthetic biology approaches in regulation of targeted gene expression. Curr Opin Plant Biol. 63:102036. 10.1016/j.pbi.2021.102036. 33930839

[kiag337-B35] Irniger S, Sanfaçon H, Egli CM, Braus GH. 1992. Different sequence elements are required for function of the cauliflower mosaic virus polyadenylation site in *Saccharomyces cerevisiae* compared with in plants. Mol Cell Biol. 12:2322–2330. 10.1128/mcb.12.5.2322-2330.1992. 1373813 PMC364404

[kiag337-B36] Jiao X et al 2018. Exchanging the order of carotenogenic genes linked by porcine teschovirus-1 2A peptide enable to optimize carotenoid metabolic pathway in Saccharomyces cerevisiae. RSC Adv. 8:34967–34972. 10.1039/C8RA06510A. 35547038 PMC9087642

[kiag337-B37] Jores T et al 2021. Synthetic promoter designs enabled by a comprehensive analysis of plant core promoters. Nat Plants. 7:842–855. 10.1038/s41477-021-00932-y. 34083762 PMC10246763

[kiag337-B38] Jozwiak A et al 2024. A cellulose synthase–like protein governs the biosynthesis of Solanum alkaloids. Science. 386:eadq5721. 10.1126/science.adq5721. 39700293

[kiag337-B39] Jung S, Maeda HA. 2024. Debottlenecking the L-DOPA 4,5-dioxygenase step with enhanced tyrosine supply boosts betalain production in *Nicotiana benthamiana*. Plant Physiol. 195:2456–2471. 10.1093/plphys/kiae166. 38498597

[kiag337-B40] Kay R, Chan A, Daly M, McPherson J. 1987. Duplication of CaMV 35S promoter sequences creates a strong enhancer for plant genes. Science. 236:1299–1302. 10.1126/science.236.4806.1299. 17770331

[kiag337-B41] Khan MI . 2016. Plant betalains: safety, antioxidant activity, clinical efficacy, and bioavailability. Compr Rev Food Sci Food Saf. 15:316–330. 10.1111/1541-4337.12185. 33371594

[kiag337-B42] Khan MI, Polturak G. 2025. Biotechnological production and emerging applications of betalains: a review. Biotechnol Adv. 81:108576. 10.1016/j.biotechadv.2025.108576. 40204005

[kiag337-B43] Klepikova AV, Kasianov AS, Gerasimov ES, Logacheva MD, Penin AA. 2016. A high resolution map of the *Arabidopsis thaliana* developmental transcriptome based on RNA-seq profiling. Plant J. 88:1058–1070. 10.1111/tpj.13312. 27549386

[kiag337-B44] Koncz C, Schell J. 1986. The promoter of TL-DNA gene 5 controls the tissue-specific expression of chimaeric genes carried by a novel type of Agrobacterium binary vector. Mol Gen Genet. 204:383–396. 10.1007/BF00331014.

[kiag337-B45] Kramer MC et al 2025. Identification of a cleaved aberrant RNA associated with the initiation of transgene silencing. Plant Cell. 37:koaf219. 10.1093/plcell/koaf219. 40973620

[kiag337-B46] Lopez-Nieves S et al 2018. Relaxation of tyrosine pathway regulation underlies the evolution of betalain pigmentation in Caryophyllales. New Phytol. 217:896–908. 10.1111/nph.14822. 28990194

[kiag337-B47] Liu W, Stewart CN. 2015. Plant synthetic biology. Trends Plant Sci. 20:309–317. 10.1016/j.tplants.2015.02.004. 25825364

[kiag337-B48] Livak KJ, Schmittgen TD. 2001. Analysis of relative gene expression data using real-time quantitative PCR and the 2^−ΔΔCT^ method. Methods. 25:402–408. 10.1006/meth.2001.1262. 11846609

[kiag337-B49] Ludwig M et al 2020. Database-independent molecular formula annotation using Gibbs sampling through ZODIAC. Nat Mach Intell. 2:629–641. 10.1038/s42256-020-00234-6.

[kiag337-B50] Maeda HA . 2019. Harnessing evolutionary diversification of primary metabolism for plant synthetic biology. J Biol Chem. 294:16549–16566. 10.1074/jbc.REV119.006132. 31558606 PMC6851331

[kiag337-B51] Maeda HA, Fernie AR. 2021. Evolutionary history of plant metabolism. Annu Rev Plant Biol. 72:185–216. 10.1146/annurev-arplant-080620-031054. 33848429

[kiag337-B52] Markel K, Sabety J, Wijesinghe S, Shih PM. 2024. Design and characterization of a transcriptional repression toolkit for plants. ACS Synth Biol. 13:3137–3143. 10.1021/acssynbio.4c00404. 39313930 PMC11494698

[kiag337-B53] Martins IR et al 2024. Betalains from vegetable peels: extraction methods, stability, and applications as natural food colorants. Food Res Int. 195:114956. 10.1016/j.foodres.2024.114956. 39277261

[kiag337-B54] McCormac AC, Elliott MC, Chen DF. 1998. A simple method for the production of highly competent cells of Agrobacterium for transformation via electroporation. Mol Biotechnol. 9:155–159. 10.1007/BF02760816. 9658392

[kiag337-B55] Nielsen J, Keasling JD. 2016. Engineering cellular metabolism. Cell. 164:1185–1197. 10.1016/j.cell.2016.02.004. 26967285

[kiag337-B56] Nirmal NP et al 2024. Betalains protect various body organs through antioxidant and anti-inflammatory pathways. Food Sci Hum Wellness. 13:1109–1117. 10.26599/FSHW.2022.9250093.

[kiag337-B57] Novais C et al 2022. Natural food colorants and preservatives: a review, a demand, and a challenge. J Agric Food Chem. 70:2789–2805. 10.1021/acs.jafc.1c07533. 35201759 PMC9776543

[kiag337-B58] Odell JT, Nagy F, Chua N-H. 1985. Identification of DNA sequences required for activity of the cauliflower mosaic virus 35S promoter. Nature. 313:810–812. 10.1038/313810a0. 3974711

[kiag337-B59] Owen C, Patron NJ, Huang A, Osbourn A. 2017. Harnessing plant metabolic diversity. Curr Opin Chem Biol. 40:24–30. 10.1016/j.cbpa.2017.04.015. 28527344 PMC5693780

[kiag337-B60] Patron NJ et al 2015. Standards for plant synthetic biology: a common syntax for exchange of DNA parts. New Phytol. 208:13–19. 10.1111/nph.13532. 26171760

[kiag337-B61] Petersen M, Collier R, Williams E. 2022. Alternative transit peptides to increase plant transformation efficiency. U.S. Patent No. US 20220389440A1.

[kiag337-B62] Polturak G, Aharoni A. 2019. Advances and future directions in betalain metabolic engineering. New Phytol. 224:1472–1478. 10.1111/nph.15973. 31148166

[kiag337-B63] Polturak G et al 2017. Engineered gray mold resistance, antioxidant capacity, and pigmentation in betalain-producing crops and ornamentals. Proc Natl Acad Sci U S A. 114:9062–9067. 10.1073/pnas.1707176114. 28760998 PMC5576821

[kiag337-B64] Pouvreau B, Vanhercke T, Singh S. 2018. From plant metabolic engineering to plant synthetic biology: the evolution of the design/build/test/learn cycle. Plant Sci. 273:3–12. 10.1016/j.plantsci.2018.03.035. 29907306

[kiag337-B65] Pramanik D, Lee K, Wang K. 2024. A simple and efficient method for betalain quantification in RUBY-expressing plant samples. Front Plant Sci. 15:1449409. 10.3389/fpls.2024.1449409. 39359623 PMC11445021

[kiag337-B66] Rizzo P, Chavez BG, Leite Dias S, D’Auria JC. 2023. Plant synthetic biology: from inspiration to augmentation. Curr Opin Biotechnol. 79:102857. 10.1016/j.copbio.2022.102857 36502769

[kiag337-B67] Sawicki T, Bączek N, Wiczkowski W. 2016. Betalain profile, content and antioxidant capacity of red beetroot dependent on the genotype and root part. J Funct Foods. 27:249–261. 10.1016/j.jff.2016.09.004.

[kiag337-B68] Schenck CA, Chen S, Siehl DL, Maeda HA. 2015. Non-plastidic, tyrosine-insensitive prephenate dehydrogenases from legumes. Nat Chem Biol. 11:52–57. 10.1038/nchembio.1693. 25402771

[kiag337-B69] Schenck CA et al 2017. Molecular basis of the evolution of alternative tyrosine biosynthetic routes in plants. Nat Chem Biol. 13:1029–1035. 10.1038/nchembio.2414. 28671678

[kiag337-B70] Schenck CA, Maeda HA. 2018. Tyrosine biosynthesis, metabolism, and catabolism in plants. Phytochemistry. 149:82–102. 10.1016/j.phytochem.2018.02.003. 29477627

[kiag337-B71] Schmid R et al 2023. Integrative analysis of multimodal mass spectrometry data in MZmine 3. Nat Biotechnol. 41:447–449. 10.1038/s41587-023-01690-2. 36859716 PMC10496610

[kiag337-B72] Schmidt GW, Delaney SK. 2010. Stable internal reference genes for normalization of real-time RT-PCR in tobacco (*Nicotiana tabacum*) during development and abiotic stress. Mol Genet Genomics. 283:233–241. 10.1007/s00438-010-0511-1. 20098998

[kiag337-B73] Selma S, Ntelkis N, Nguyen TH, Goossens A. 2023. Engineering the plant metabolic system by exploiting metabolic regulation. Plant J. 114:1149–1163. 10.1111/tpj.16157. 36799285

[kiag337-B74] Soares A et al 2014. The role of L-DOPA in plants. Plant Signal Behav. 9:e28275. 10.4161/psb.28275. 24598311 PMC4091518

[kiag337-B75] Stintzing FC, Schieber A, Carle R. 2003. Evaluation of colour properties and chemical quality parameters of cactus juices. Eur Food Res Technol. 216:303–311. 10.1007/s00217-002-0657-0.

[kiag337-B76] Strobbe S, Verstraete J, Stove C, Van Der Straeten D. 2021. Metabolic engineering provides insight into the regulation of thiamin biosynthesis in plants. Plant Physiol. 186:1832–1847. 10.1093/plphys/kiab198. 33944954 PMC8331165

[kiag337-B77] Sweetlove LJ, Fernie AR. 2013. The spatial organization of metabolism within the plant cell. Annu Rev Plant Biol. 64:723–746. 10.1146/annurev-arplant-050312-120233. 23330793

[kiag337-B78] Tan J et al 2025. eRUBY rice: co-expression of a feedback-insensitive TyrA arogenate dehydrogenase with RUBY enhances endosperm betalain levels. Plant Physiol. 199:kiaf416. 10.1093/plphys/kiaf416. 41055326

[kiag337-B79] Thomsen PT et al 2023. Beet red food colourant can be produced more sustainably with engineered *Yarrowia lipolytica*. Nat Microbiol. 8:2290–2303. 10.1038/s41564-023-01517-5. 38030899 PMC10686825

[kiag337-B80] Timoneda A et al 2018. Redirecting primary metabolism to boost production of tyrosine-derived specialised metabolites in planta. Sci Rep. 8:17256. 10.1038/s41598-018-33742-y. 30467357 PMC6250739

[kiag337-B81] Towards FnB . 2025. Global food colors market: industry trends, growth drivers, and future outlook. https://www.towardsfnb.com/insights/food-colors-market.

[kiag337-B82] U.S. Food and Drug Administration . 2025a. FD&C Red No. 3. https://www.fda.gov/industry/color-additives/fdc-red-no-3.

[kiag337-B83] U.S. Food and Drug Administration . 2025b. HHS and FDA to phase out petroleum-based synthetic dyes from Nation's Food Supply. https://www.fda.gov/news-events/press-announcements/hhs-fda-phase-out-petroleum-based-synthetic-dyes-nations-food-supply.

[kiag337-B84] Vancanneyt G, Schmidt R, O’Connor-Sanchez A, Willmitzer L, Rocha-Sosa M. 1990. Construction of an intron-containing marker gene: splicing of the intron in transgenic plants and its use in monitoring early events in Agrobacterium-mediated plant transformation. Mol Gen Genet. 220:245–250. 10.1007/BF00260489. 2325623

[kiag337-B85] Wang Y, Demirer GS. 2023. Synthetic biology for plant genetic engineering and molecular farming. Trends Biotechnol. 41:1182–1198. 10.1016/j.tibtech.2023.03.007. 37012119

[kiag337-B86] Weber E, Engler C, Gruetzner R, Werner S, Marillonnet S. 2011. A modular cloning system for standardized assembly of multigene constructs. PLoS One. 6:e16765. 10.1371/journal.pone.0016765. 21364738 PMC3041749

[kiag337-B87] Werner S, Engler C, Weber E, Gruetzner R, Marillonnet S. 2012. Fast track assembly of multigene constructs using Golden Gate cloning and the MoClo system. Bioengineered. 3:38–43. 10.4161/bbug.3.1.18223. 22126803

[kiag337-B88] Xiao C, Zhou G, He T, Li C. 2025. Transport of secondary metabolites in plants: mechanistic insights and transporter engineering for crop improvement. Plant Commun. 6:101536. 10.1016/j.xplc.2025.101536. 41013897 PMC12744758

[kiag337-B89] Xue Y et al 2026. Development of eRUBY maize with betalain-enriched endosperm using a push-and-pull synthetic metabolic engineering strategy. Crop J. 14:129–140. 10.1016/j.cj.2025.10.005.

[kiag337-B90] Yang J-S, Reyna-Llorens I. 2023. Plant synthetic biology: exploring the frontiers of sustainable agriculture and fundamental plant biology. J Exp Bot. 74:3787–3790. 10.1093/jxb/erad220. 37462736

[kiag337-B91] Yaschenko AE, Fenech M, Mazzoni-Putman S, Alonso JM, Stepanova AN. 2022. Deciphering the molecular basis of tissue-specific gene expression in plants: can synthetic biology help? Curr Opin Plant Biol. 68:102241. 10.1016/j.pbi.2022.102241. 35700675 PMC10605770

[kiag337-B92] Yau Y-Y, Easterling M, Brennan L. 2020. Rapid Agrobacterium-mediated transformation of tobacco cotyledons using toothpicks. In: Kumar A, Yau Y-Y, Ogita S, Scheibe R, editors. Climate Change, Photosynthesis and Advanced Biofuels. Springer. p. 407–423.

[kiag337-B93] Yobi A, Angelovici R. 2018. A high-throughput absolute-level quantification of protein-bound amino acids in seeds. Curr Protoc Plant Biol. 3:e20084. 10.1002/cppb.20084. 30408333

[kiag337-B94] Yokoyama R, de Oliveira MVV, Kleven B, Maeda HA. 2021. The entry reaction of the plant shikimate pathway is subjected to highly complex metabolite-mediated regulation. Plant Cell. 33:671–696. 10.1093/plcell/koaa042. 33955484 PMC8136874

[kiag337-B95] Zhang N, McHale LK, Finer JJ. 2015. Isolation and characterization of “GmScream” promoters that regulate highly expressing soybean (*Glycine max* Merr.) genes. Plant Sci. 241:189–198. 10.1016/j.plantsci.2015.10.010. 26706070

[kiag337-B96] Zhao C et al 2018. Co-compartmentation of terpene biosynthesis and storage via synthetic droplet. ACS Synth Biol. 7:774–781. 10.1021/acssynbio.7b00368. 29439563

[kiag337-B97] Zhou A et al 2023. A suite of constitutive promoters for tuning gene expression in plants. ACS Synth Biol. 12:1533–1545. 10.1021/acssynbio.3c00075. 37083366

[kiag337-B98] Zhu X et al 2021. Synthetic biology of plant natural products: from pathway elucidation to engineered biosynthesis in plant cells. Plant Commun. 2:100229. 10.1016/j.xplc.2021.100229. 34746761 PMC8553972

