# Supplementary material for: Chassis selection and metabolic fine-tuning enable efficient in planta betalain production
Source: Plant Physiol. 2026 Jun 8;201(3):kiag337. doi: 10.1093/plphys/kiag337 (PMC13368612; doi:10.1093/plphys/kiag337)
Supplement: kiag337_Supplementary_Data [file kiag337_supplementary_data.zip › kiag337_Supplementary_Data.pdf]

Supplemental Data

Chassis Selection and Metabolic Fine-Tuning Enable Efficient *in planta* Betalain Production

Soyoung Jung[a], Marcos V. V. de Oliveira[a], Ray Collier[b], Abou Yobi[c], Ruthie Angelovici[c], Shawn M. Kaeppler[b], Hiroshi A. Maeda[a]

[a]Department of Botany, University of Wisconsin-Madison, Madison, WI, USA
[b]Wisconsin Crop Innovation Center, University of Wisconsin-Madison, Middleton, WI, USA
[c]Cristopher S. Bond Life Sciences Center, Division of Biological Sciences, Interdisciplinary Plant Group, University of Missouri, Columbia, MO, USA

Supplemental Figures

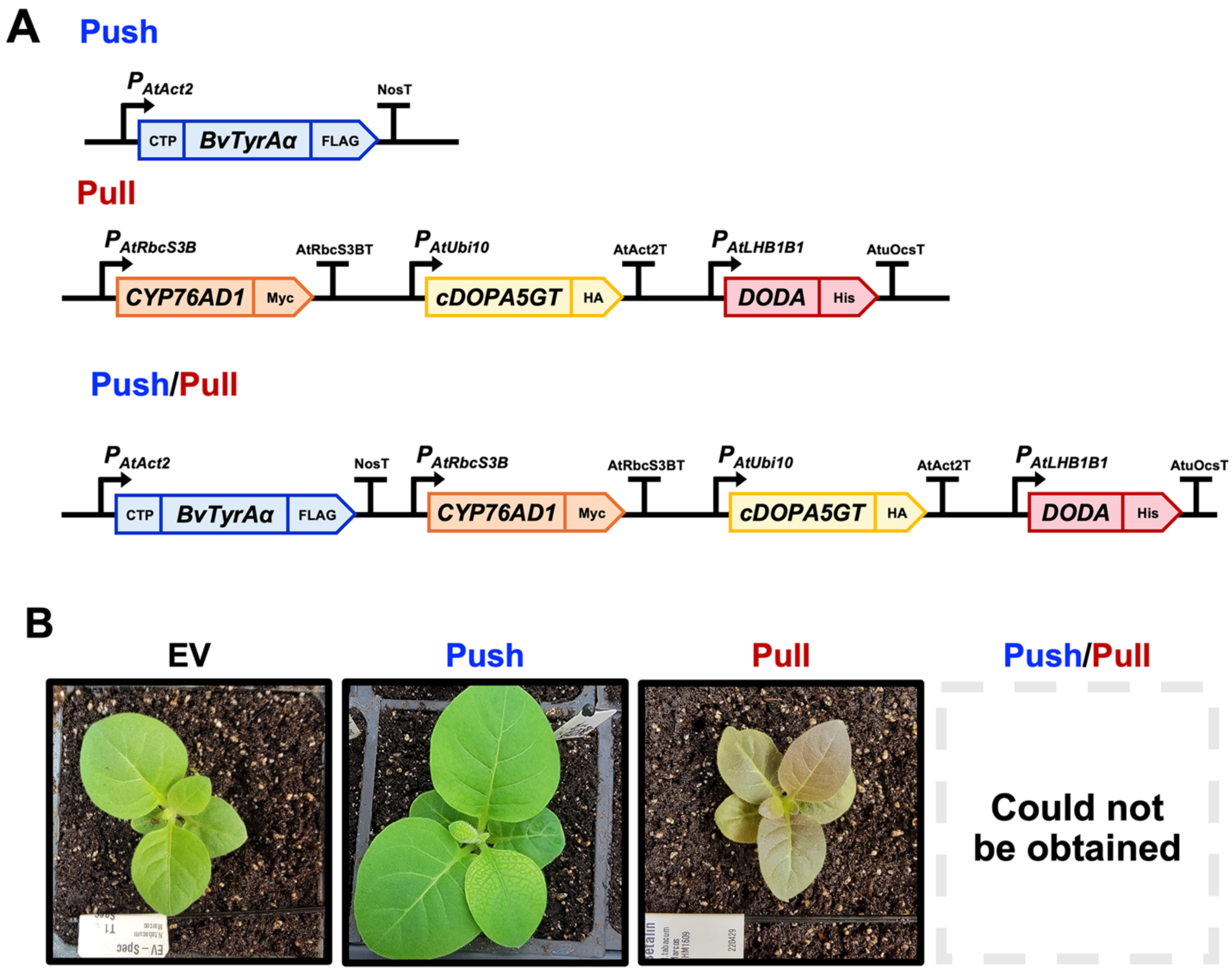

Figure S1. Previous push/pull construct failed to generate stable transgenic lines
(A) Schematic of initial push, pull, and push/pull constructs. Arabidopsis Actin2 promoter (P_{AtAct2}) and nopaline synthase terminator (NosT) were used for expressing *BvTyrAα* coding sequence flanked with N-terminal petunia chloroplast transit peptide (CTP) and C-terminal FLAG tag; Arabidopsis Rubisco small subunit 3B promoter ($P_{AtRbcS3B}$) and terminator (AtRbcS3BT) were used for expressing *BvCYP76AD1* coding sequence flanked with C-terminal Myc tag; Arabidopsis Ubiquitin10 promoter ($P_{AtUbi10}$) and Arabidopsis Actin2 terminator (AtAct2T) were used for expressing *MjcDOPA5GT* coding sequence flanked with C-terminal HA tag; Arabidopsis light-harvesting chlorophyll-protein complex II subunit B1 promoter ($P_{AtLHB1B1}$) and *Agrobacterium tumefaciens* octopine synthase terminator (AtuOcsT) were used for expressing *BvDODA* flanked with C-terminal His tag. **(B)** Tissue culture images of tobacco (*N. tabacum*) show failure to regenerate shoots with the initial push/pull construct. EV, empty vector.

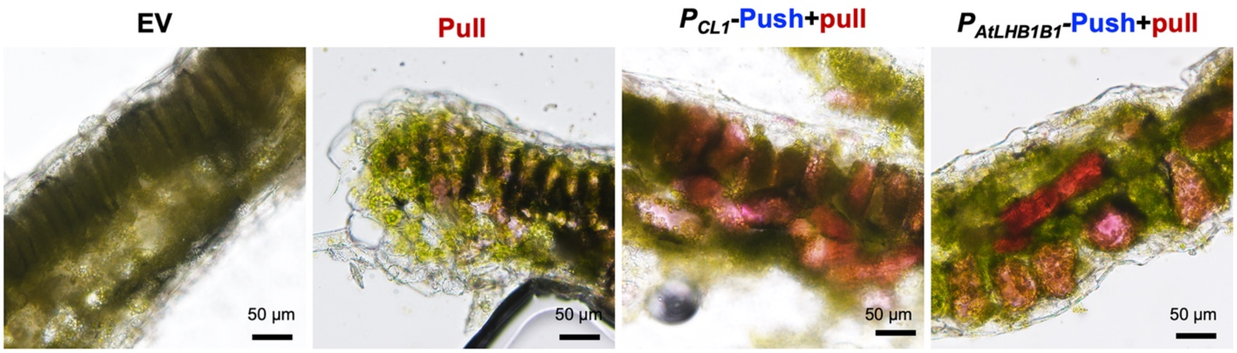

Figure S2. Betalains are stored mainly in central vacuoles of mesophyll cells in *N. tabacum*
Compound microscopy images with cross-section of T_0 transgenic tobacco leaves expressing betalain biosynthetic genes show red pigment accumulation primarily in the central vacuoles of mesophyll cells. EV, empty vector.

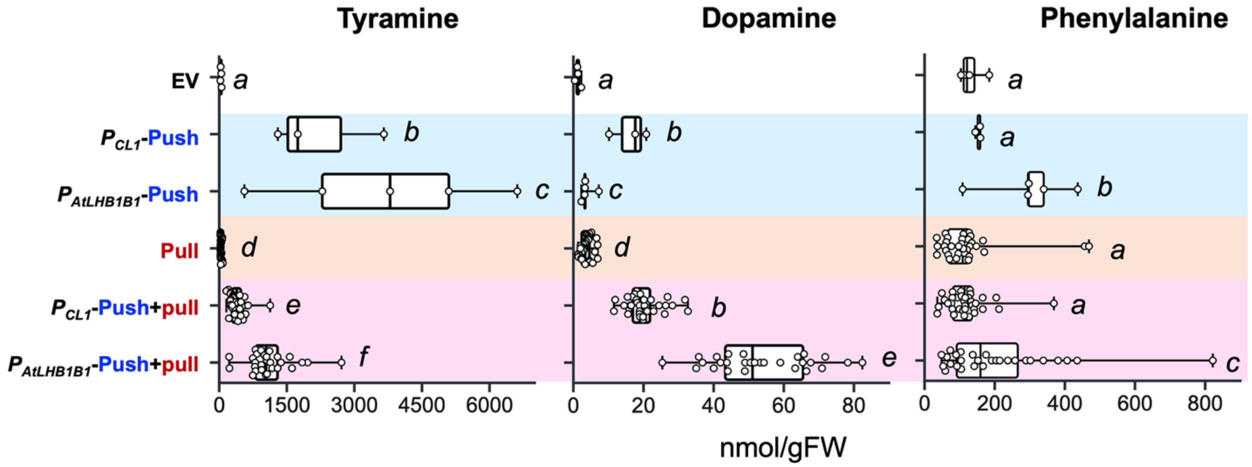

Figure S3. Targeted metabolite analysis of tyrosine derivatives in leaves of T_0 tobacco lines. Quantification of tyramine, dopamine, and phenylalanine levels in T_0 stable tobacco transgenic lines using LC-MS. Leaf discs were collected at six-week after transplanting from rooting media to soil for metabolite analysis. The experiment was conducted using biological replicates, with sample sizes as follows: EV (n = 4), P_{CL1}-push (n = 3), $P_{AtLHB1B1}$-push (n = 5), pull (n = 32), P_{CL1}-push+pull (n = 34), $P_{AtLHB1B1}$-push+pull (n = 28). Letters denote significant differences based on one-way ANOVA of $\log_{10}$-transformed values followed by Tukey's HSD test ($p < 0.05$). EV, empty vector. Box plots were generated in Biorender (https://www.biorender.com).

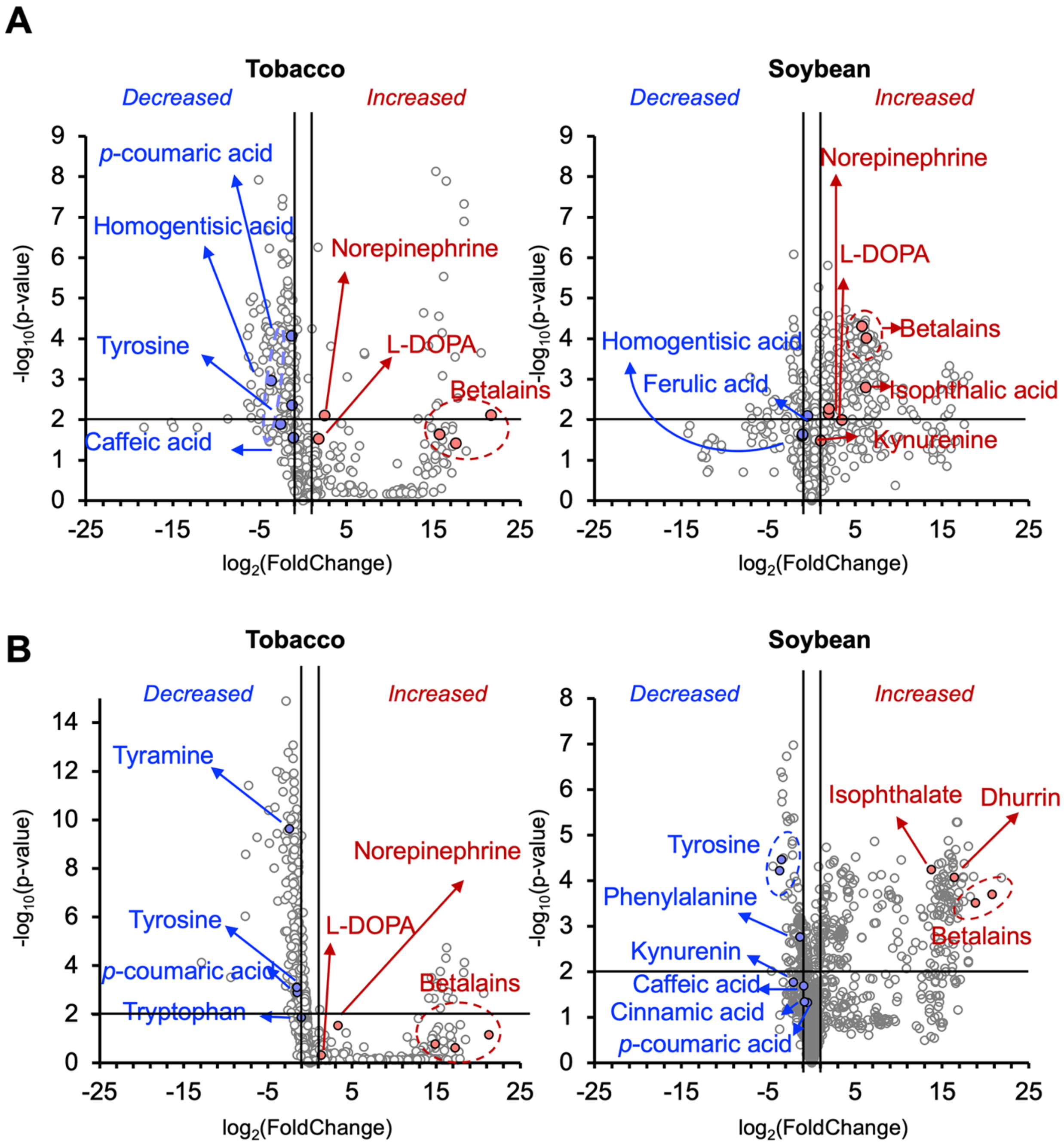

Figure S4. Untargeted metabolomics reveal candidate metabolites altered in leaves of transgenic tobacco and soybean T_0 lines

Volcano plots comparing the untargeted metabolomics data of **(A)** $P_{AtLHB1B1}$-push+pull lines compared to $P_{AtLHB1B1}$-push lines and **(B)** P_{CL1}-push+pull lines compared to P_{CL1}-push lines in either tobacco or soybean T_0 lines. Horizontal lines correspond to a $p < 0.01$ according to Student's t-test (two-tailed test, equal variance). Vertical lines correspond to a fold change of ≥2 times.

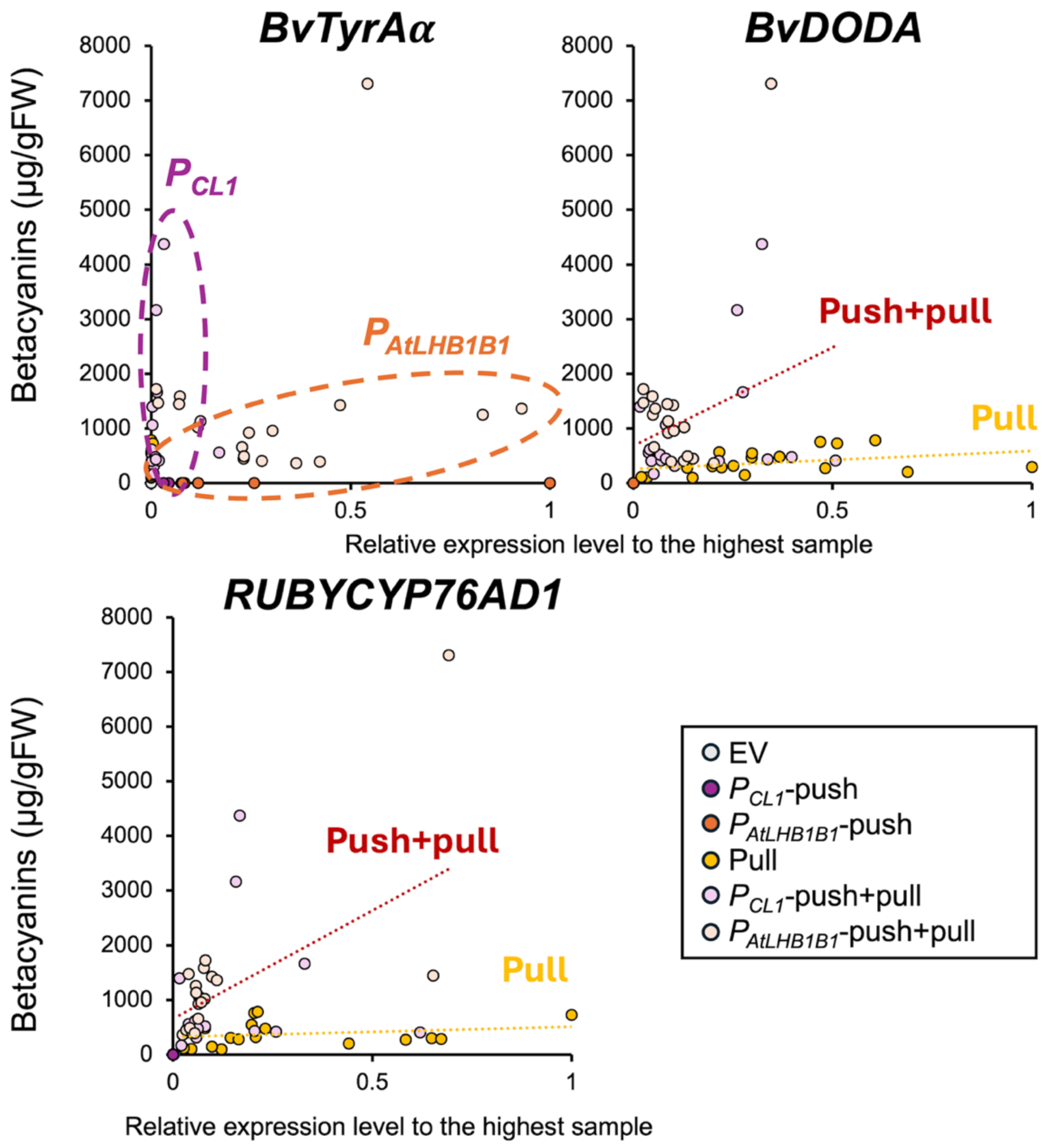

Figure S5. Transcript analysis in T_0 tobacco transgenic lines using RT-qPCR show efficient production of betalains in push+pull lines compared to the pull lines

Transcript levels of each transgene (i.e., *BvTyrAα, BvDODA* and *RUBYCYP76AD1*) were analyzed by RT-qPCR in the leaf samples from independent T_0 tobacco transgenic lines and plotted against their betacyanin contents. Expression of the *NtPP2A* gene was used as an internal reference to normalize the sample-to-sample variations among cDNA preparations. P_{CL1}-driven constructs showed lower *BvTyrAα* expression than $P_{AtLHB1B1}$-conterparts. The majenta dashed circle indicates *BvTyrAα* transcript abundance in P_{CL1}-driven push or push+pull lines whereas the orange dashed circle highlights abundance in $P_{AtLHB1B1}$-driven counterparts. Red and yellow dotted lines represent linear trendline illustrating the correlations between gene expression and betacyanin accumulation in push+pull and pull lines, respectively. EV, empty vector.

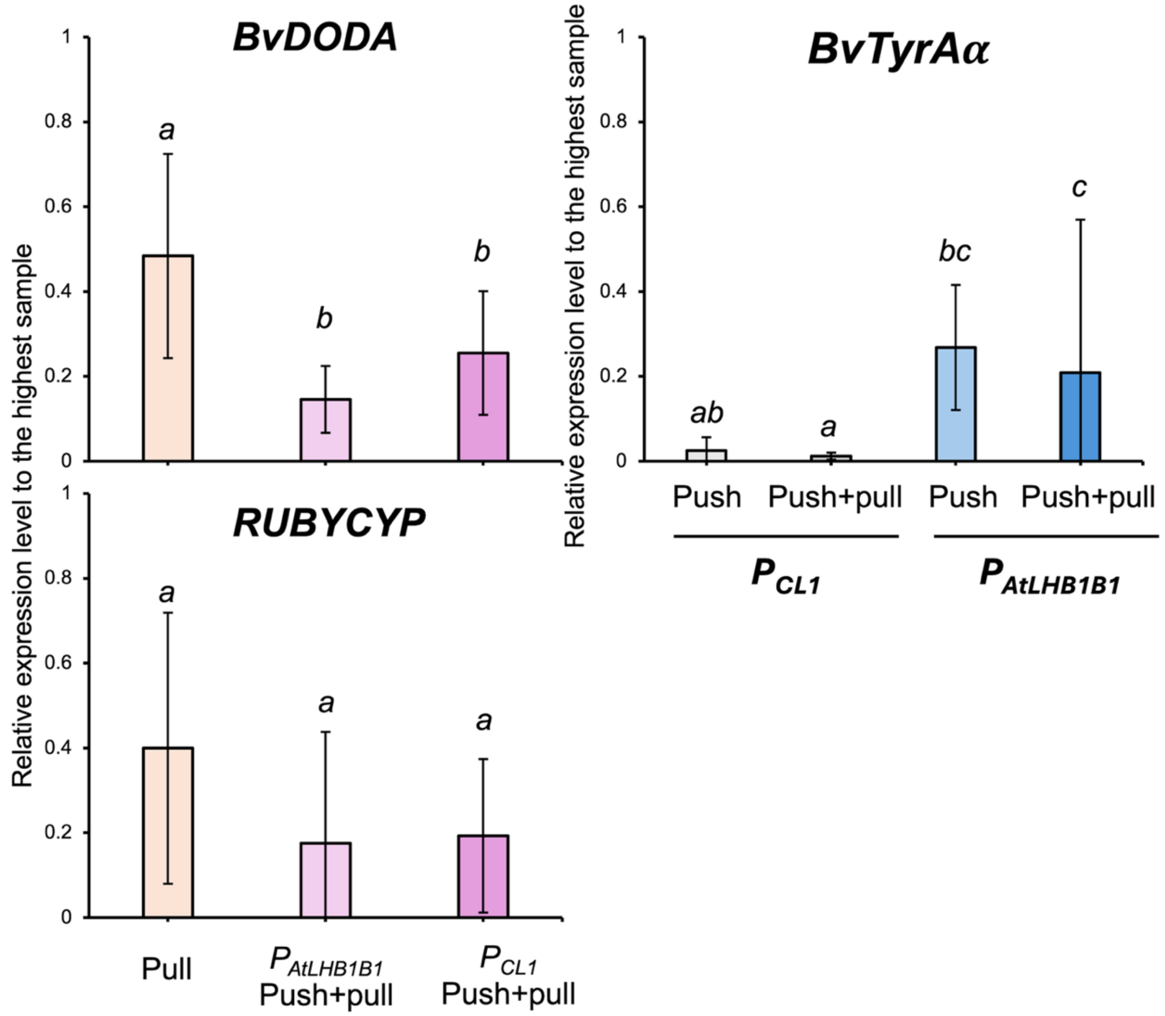

Figure S6. Expression of betalain genes is similar or even higher in tobacco transgenic T_0 pull lines compared to push+pull lines

Transcript levels of each transgene (i.e., *BvTyrAα, BvDODA* and *RUBYCYP76AD1*) were analyzed by RT-qPCR in the leaf samples from independent T_0 tobacco transgenic lines. Expression of the *NtPP2A* gene was used as an internal reference to normalize the sample-to-sample variations among cDNA preparations. Bars represent mean value of the transcript levels, with error bars representing standard deviation. The experiment was conducted using biological replicates, with sample sizes as follows: P_{CL1}-push (n = 3), $P_{AtLHB1B1}$-push (n = 5), pull (n = 32), P_{CL1}-push+pull (n = 34), $P_{AtLHB1B1}$-push+pull (n = 28). Different letters indicate significant differences based on one-way ANOVA followed by Tukey's HSD test ($p < 0.05$).

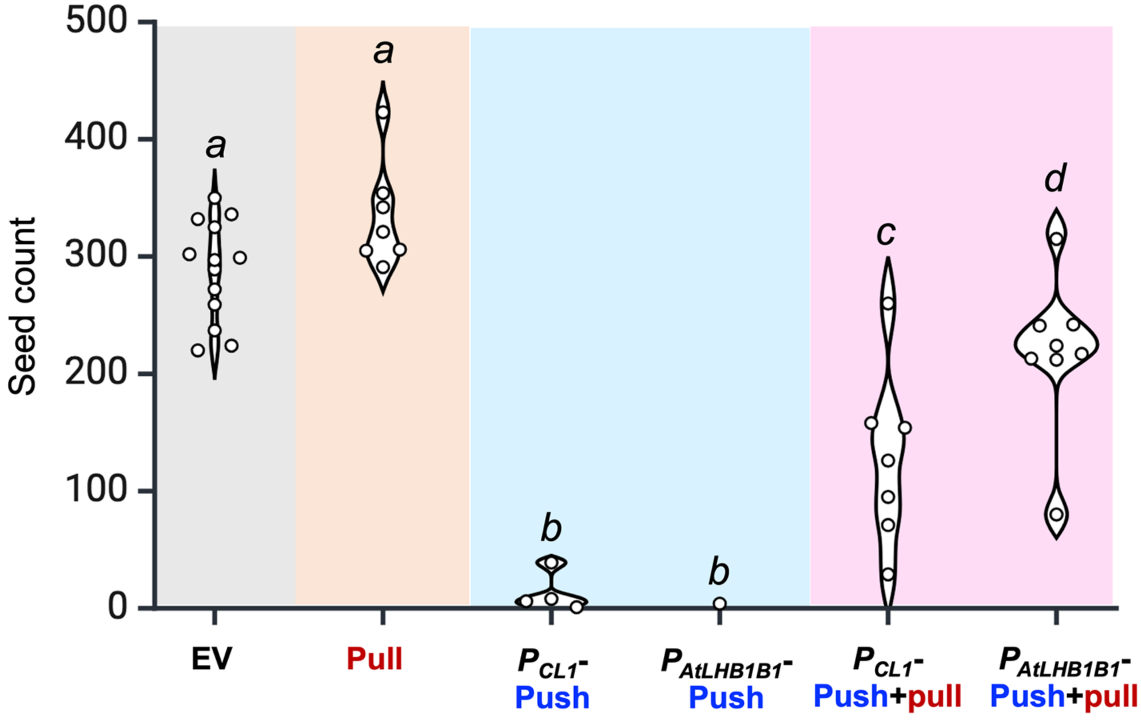

Figure S7. Push+pull construct partially rescue low seed yield observed in push lines in soybean T_0 plants

Seed count from single-copy inserted soybean T_0 lines expressing different constructs. Letters denote significant differences based on one-way ANOVA followed by Tukey's HSD test ($p < 0.05$). EV, empty vector. Violin plot was generated in Biorender (https://www.biorender.com).

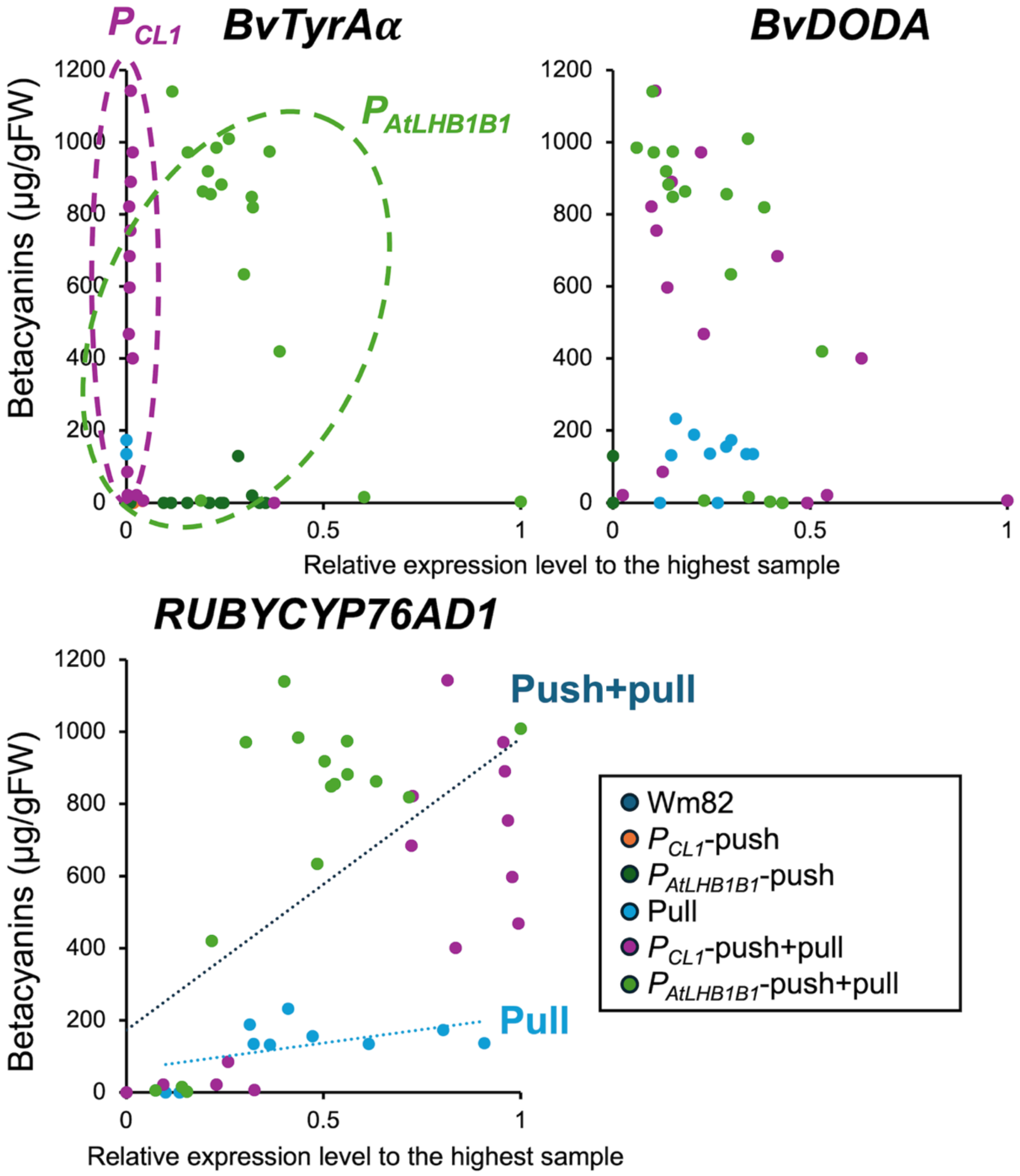

Figure S8. Transcript analysis in T_0 soybean transgenic lines using RT-qPCR show efficient production of betalains in push+pull lines compared to the pull lines.

Transcript levels of each transgene (i.e., *BvTyrAα, BvDODA* and *RUBYCYP76AD1*) were analyzed by RT-qPCR in the leaf samples from independent T_0 soybean transgenic lines and plotted against their betacyanin contents. Expression of the *GmUbi3* gene was used as an internal reference to normalize the sample-to-sample variations among cDNA preparations. P_{CL1}-driven constructs showed lower *BvTyrAα* expression than $P_{AtLHB1B1}$-conterparts. The majenta dashed circle indicates *BvTyrAα* transcript abundance in P_{CL1}-driven push or push+pull lines whereas the green dashed circle highlights abundance in $P_{AtLHB1B1}$-driven counterparts. Dark blue and light blue dotted lines represent linear trendline illustrating the correlations between gene expression and betacyanin accumulation in push+pull and pull lines, respectively. Wm82, Williams 82.

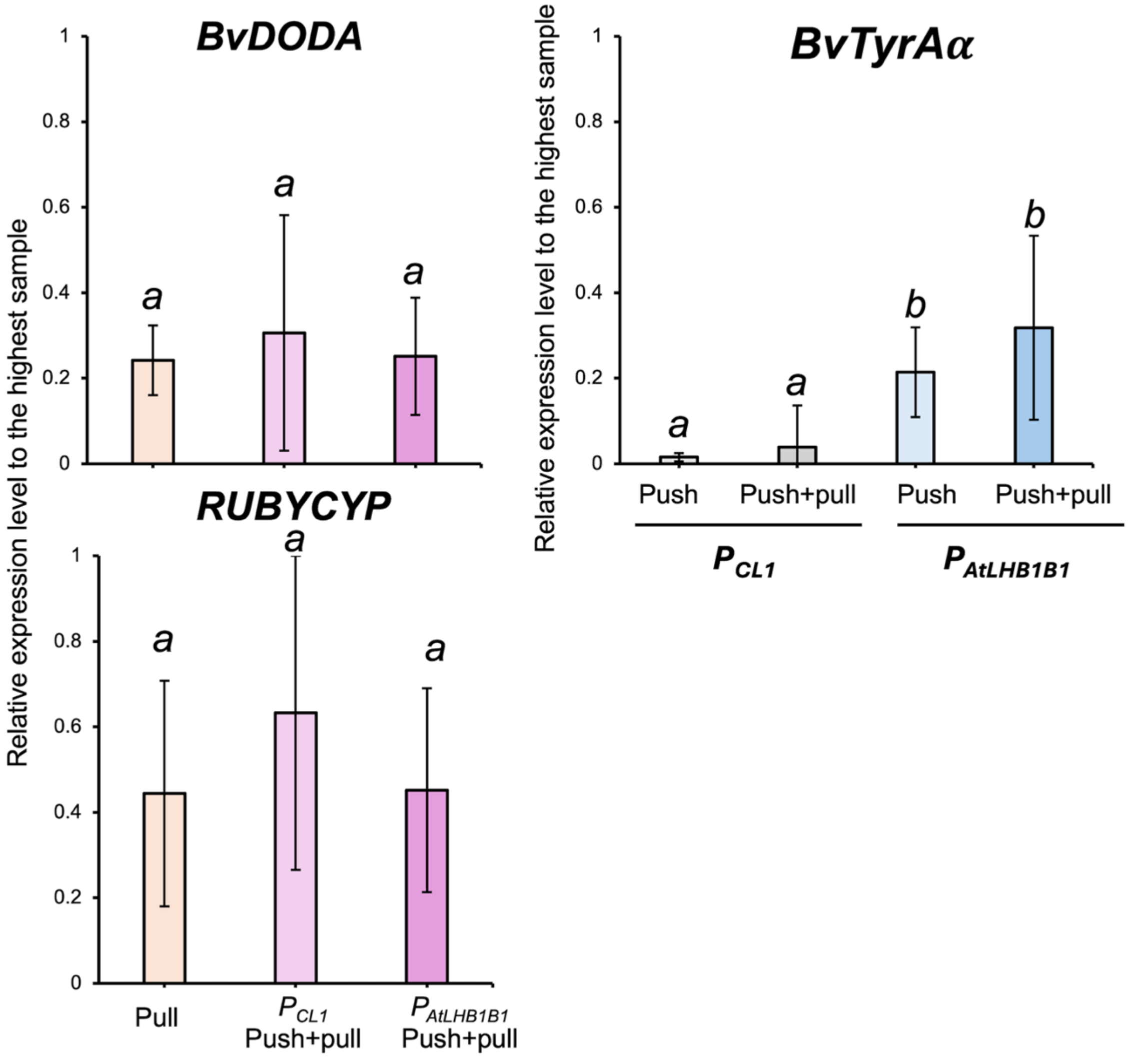

Figure S9. Expression of betalain genes does not differ in the leaves of soybean transgenic T_0 pull lines compared to push+pull lines

Transcript levels of each transgene (i.e., *BvTyrAα, BvDODA* and *RUBYCYP76AD1*) were analyzed by RT-qPCR in the leaf samples from independent T_0 soybean transgenic lines. Expression of the *GmUbi3* gene was used as an internal reference to normalize the sample-to-sample variations among cDNA preparations. Bars represent mean value of the transcript levels, with error bars representing standard deviation. The experiment was conducted using biological replicates, with sample sizes as follows: Wm82 (n = 4), P_{CL1}-push (n = 15), $P_{AtLHB1B1}$-push (n = 14), pull (n = 15), P_{CL1}-push+pull (n = 16), $P_{AtLHB1B1}$-push+pull (n = 22). Different letters indicate significant differences based on one-way ANOVA followed by Tukey's HSD test ($p < 0.05$).

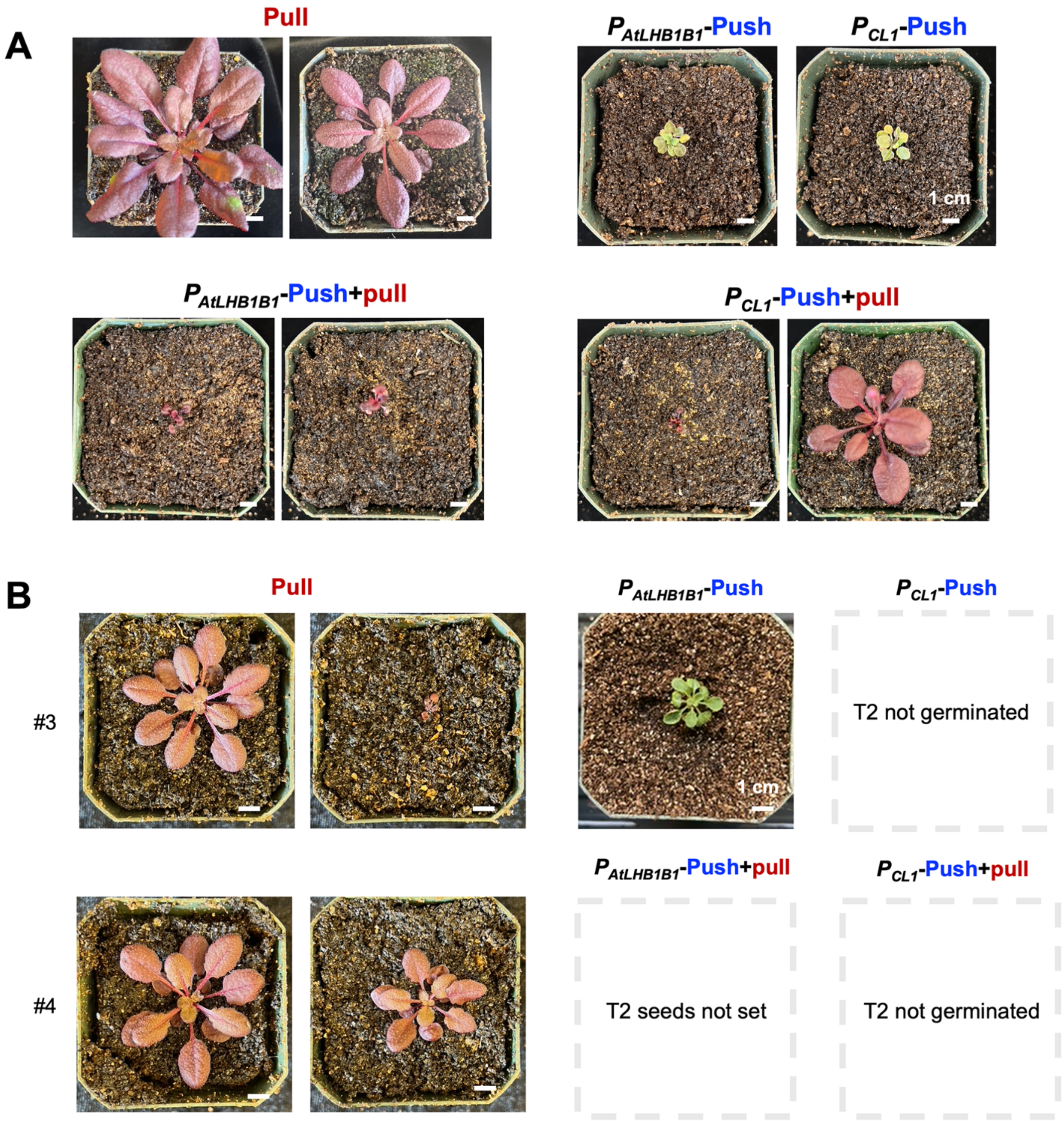

Figure S10. Arabidopsis stable transgenic lines with push+pull constructs fail to produce T_2 seeds.

(A) T_1 generation of transgenic Arabidopsis lines. Only RFP+ seeds were selected and germinated in various lines. **(B)** T_2 generation of transgenic Arabidopsis lines. The lines shown here are all single-copy inserted lines that were selected based on the segregation ratio of RFP+ and RFP- seeds.

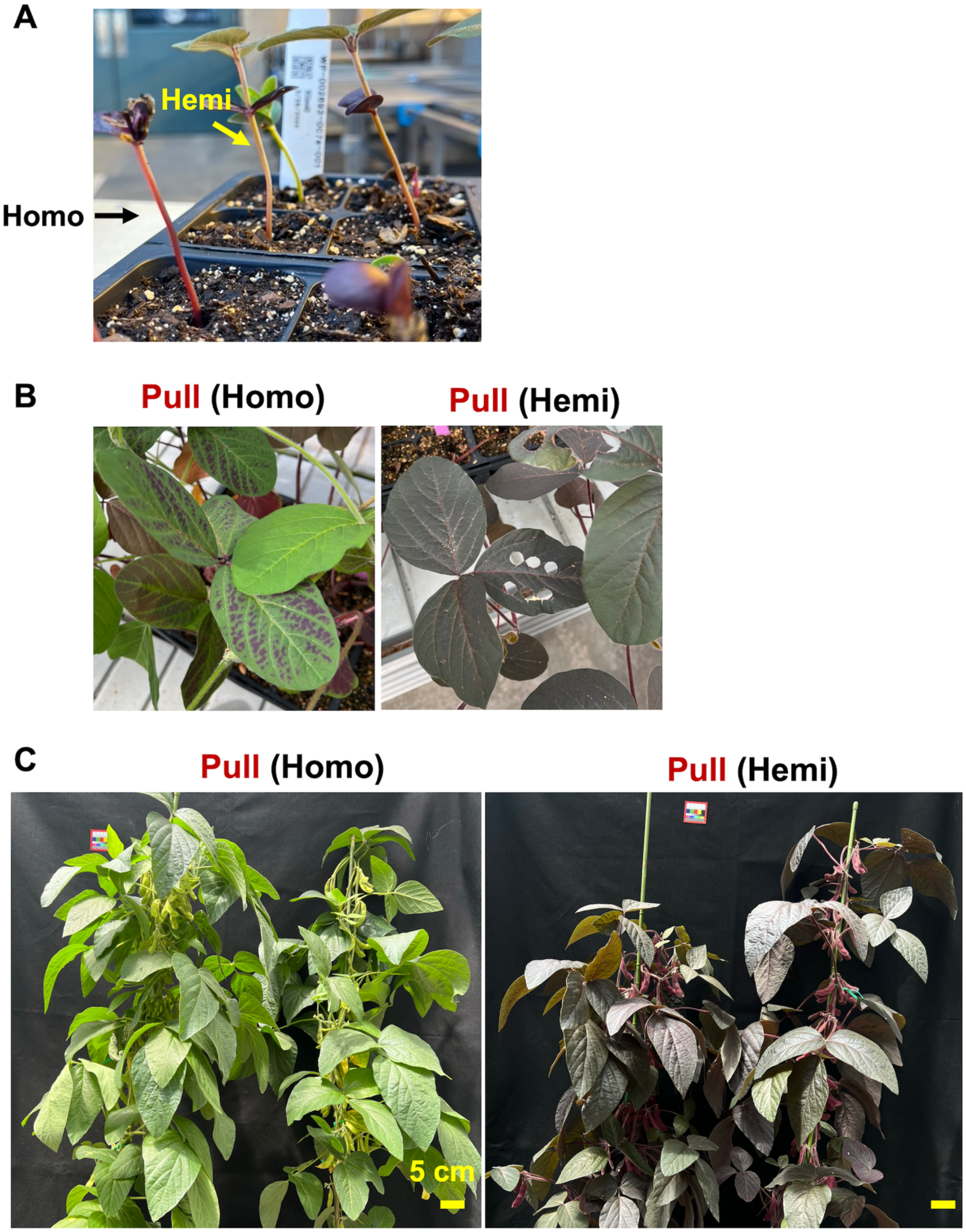

Figure S11. Pigmentation gradually fades out in T_1 homozygous pull lines
(A) Phenotypes of hemizygous or hemizygous lines of stable T_1 soybean seedlings expressing pull constructs together. Homo, homozygous; Hemi, hemizygous. **(B)** Phenotypes of fully expanded leaves in hemizygous or homozygous lines of stable T_1 soybean expressing pull constructs. Homo, homozygous; Hemi, hemizygous. **(C)** Phenotypes of hemizygous or homozygous lines of fully mature stable T_1 soybean lines expressing pull constructs together. Homo, homozygous; Hemi, hemizygous.

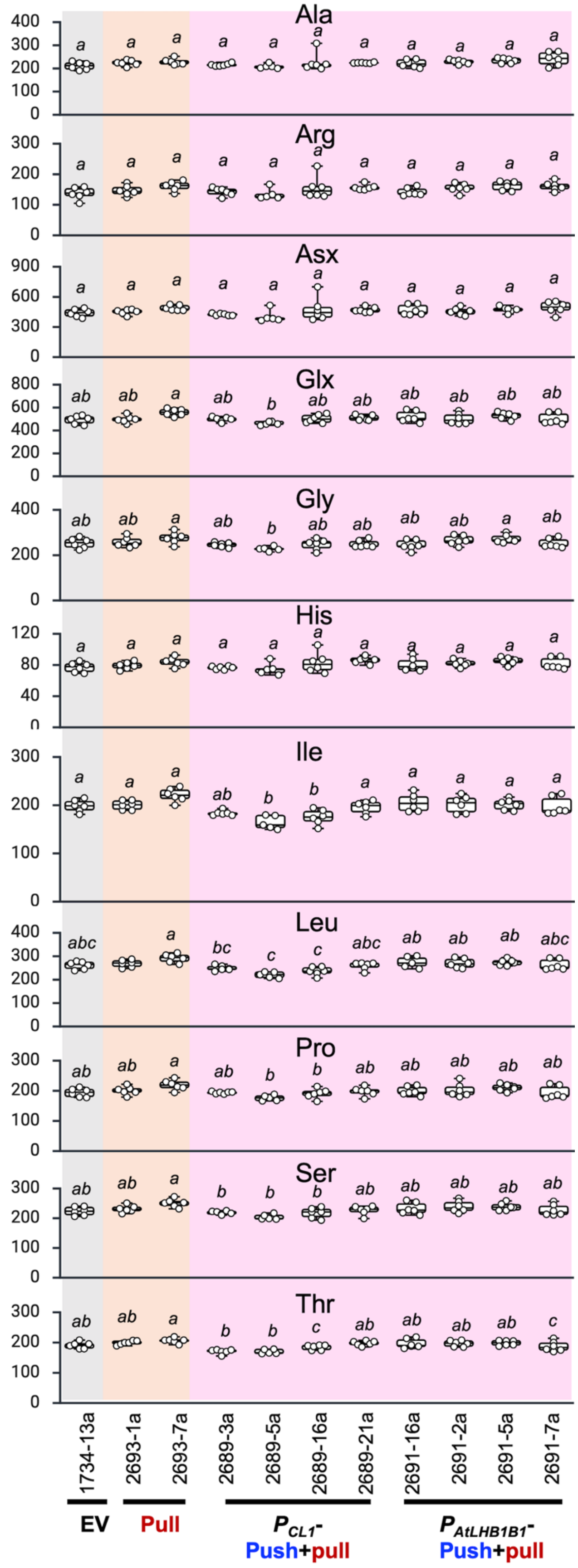
Ala
Arg
Asx
Glx
Gly
His
Ile
Leu
Pro
Ser
Thr
1734-13a
2693-1a
2693-7a
2689-3a
2689-5a
2689-16a
2689-21a
2691-16a
2691-2a
2691-5a
2691-7a
EV
Pull
PCL1-
Push+pull
PAtLHB1B1-
Push+pull

Figure S12. Protein-bound amino acid levels did not show significant increase in P_{CL1}-push+pull T_1 soybean seeds.

Quantification of protein-bound amino acid levels in soybean transgenic T_1 seeds using LC-MS. The experiment was conducted using six biological replicates. Letters indicate significant differences based on one-way ANOVA followed by Tukey's HSD test ($p < 0.05$). Box plots were generated in BioRender (https://www.biorender.com).

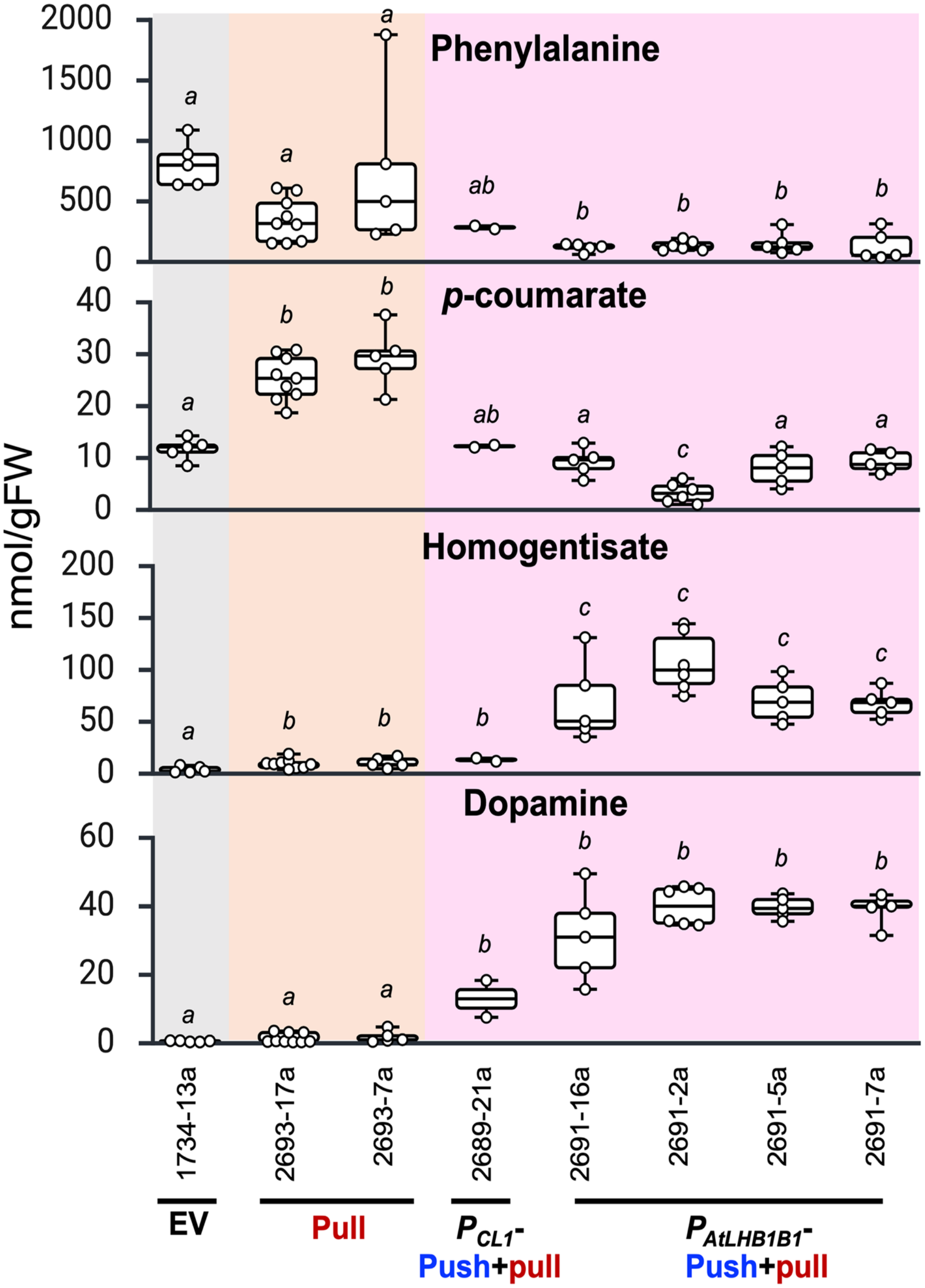

Figure S13. Targeted metabolite analysis of tyrosine derivatives and phenylpropanoids in leaves of T_1 soybean lines.

Quantification of phenylalanine, *p*-coumarate, homogentisate and dopamine levels in mature leaves of T_1 soybean stable transgenic lines using LC-MS. The experiment was conducted using biological replicates, with sample sizes as follows: EV (n = 5), 2693-17a (n = 9), 2693-7a (n = 5), 2689-21a (n = 2), 2691-16a (n = 5), 2691-2a (n = 6), 2691-5a (n = 5), 2691-7a (n = 5). Letters denote significant differences based on one-way ANOVA of log_{10}-transformed values followed by Tukey's HSD test ($p < 0.05$). EV, empty vector. Box plots were generated in Biorender (https://www.biorender.com).

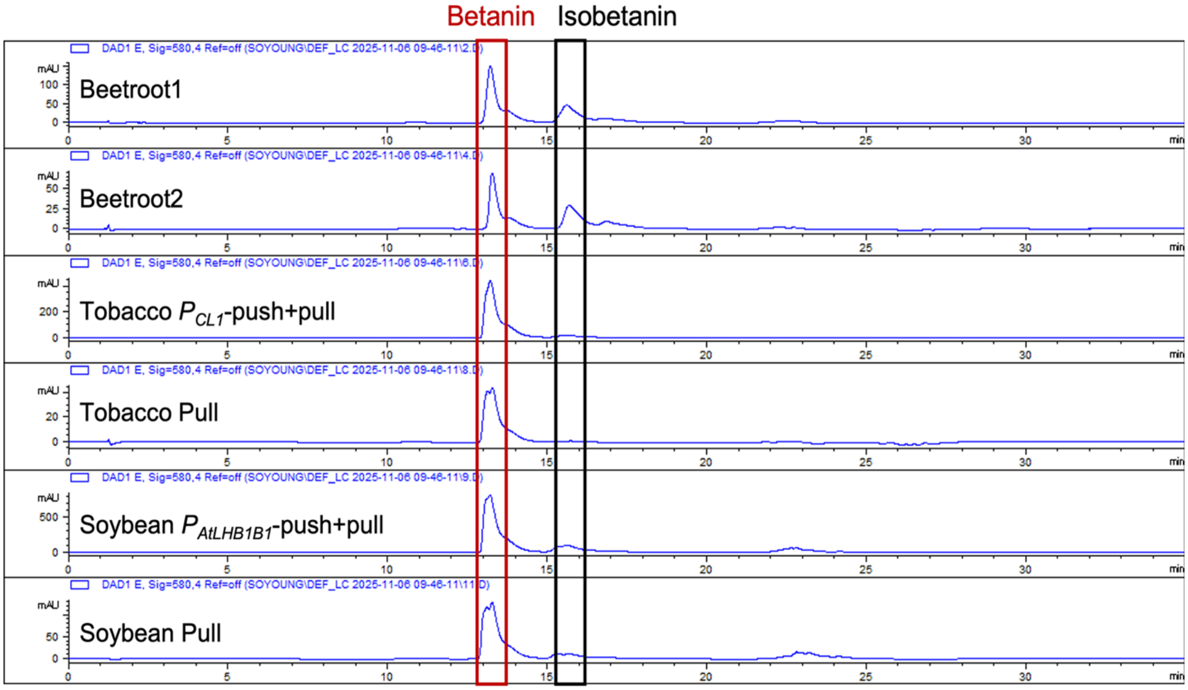

Figure S14. Transgenic tobacco and soybean lines show higher proportion of betanin to isobetanin than beetroot extract.

High-performance liquid chromatography (HPLC) chromatograms of metabolite extracts from beetroot, mature leaves of transgenic tobacco (T_1) and transgenic soybean (T_1). Absorbance profiles at 580 nm are shown. The major betacyanin peaks corresponding to betanin and isobetanin are indicated.

Supplemental Table

Table S1. Primers used in this study

Primer name	Sequence (5' - 3')	Purpose
pHM3495	GTGAAGCTGTAGGGCCTGAGC	RT-qPCR (NtPP2A)
pHM3496	CATAGGCAGGCACCAAATCC	
pHM3528	GTGTAATGTTGGATGTGTTCCC	RT-qPCR (GmUbi3)
pHM3529	ACACAATTGAGTTCAACACAAACCG	
pHM0003	CATTGGTTCAGGAAGTGCAA	RT-qPCR (BvDODA)
pHM0004	CCTTTGATTCATGGCTTCGT	
pHM0399	TTTAGGAATGCCGGGTGTAG	RT-qPCR (BvTyrAa)
pHM0400	TCTCCAAAACTCGTCCCATC	
pHM2910	GAGAGACTCGCCCCAGATTCCT	RT-qPCR (RUBYCYP76AD1)
pHM2911	CTCGCCCATCGTCAGCTCGTTC	
pHM2912	TCCGGCCACTGGGAGACAGTGA	RT-qPCR (RUBYDODA)
pHM2913	CTTGAACTGGTACATGGCGGCT	
pHM2914	CTTATGGCACATCCGCCTACGT	RT-qPCR (RUBYcDOPA6GT)
pHM2915	TATGGTTCTCTGGGAAGCCTGG	
pHM3219	GCGCgaagacGCggagTTTCAAGGCTTAGCTTAGACAAA	lv0 cloning of PCL1
pHM3220	GCGCgaagacGCAAGAGTTCTCCACATCATCATAATCATGGGA	
pHM3221	GCGCgaagacGCTCTTCCACGAGCAGCACCTG	
pHM3261	GCGCgaagacGCatggTATCTCTCTCTCGAAACCCTAGATTCC	
